# Venus flytraps' metabolome analysis discloses the metabolic fate of prey animal foodstock

**DOI:** 10.1111/tpj.70391

**Published:** 2025-08-04

**Authors:** Ines Kreuzer, Federico Scossa, Takayuki Tohge, Alisdair R. Fernie, Rainer Hedrich

**Affiliations:** ^1^ Molecular Plant Physiology & Biophysics University of Wuerzburg Julius‐von‐Sachs‐Platz 2 D‐97082 Wuerzburg Germany; ^2^ Max‐Planck Institut für Molekulare Pflanzenphysiologie Am Mühlenberg 1 D‐14476 Potsdam‐Golm Germany; ^3^ Council for Agricultural Research and Economics (CREA) Research Centre for Genomics and Bioinformatics (CREA‐GB) Via Ardeatina 546 00178 Rome Italy; ^4^ Graduate School of Biological Science Nara Institute of Science and Technology (NAIST) Ikoma 630‐0192 Japan; ^5^ Faculty of Synthetic Biology Shenzhen University of Advanced Technology (SUAT) No. 1 Gongchang Road Shenzen 518107 China

**Keywords:** Acheta domesticus, amino acid metabolism, *Dionaea muscipula*, metabolomics, plant carnivory, prey digestion, Venus flytrap

## Abstract

Carnivorous plants such as the Venus flytrap *Dionaea muscipula* survive in nutrient‐poor habitats by attracting and consuming animals. Upon deflection of the touch‐sensitive trigger hairs, the trap closes instantly. Panicking prey repeatedly collides with trigger hairs, which activate the endocrine system: mechano‐ and chemosensors translate the information on the prey's nature, size, and activity into jasmonate‐dependent lytic enzyme secretion. This digestive fluid gradually degrades its exoskeleton and internal tissues. The released substances are absorbed by glands covering the inner trap surface. To understand *Dionaea*'s modification of metabolism upon prey consumption, we compared the metabolic profiles associated with secretion and insect feeding. In favor of digestive enzyme secretion, the abundance of most amino acids decreased after JA‐stimulation without prey present. By contrast, insect feeding resulted in an increase in almost all amino acids within the trap. In agreement with the export of prey‐derived nitrogen, the abundance of certain amino acids also increased in the petiole. In response to feeding with urea, chitin, nucleic acids, or phospholipids, the amino acid profile remained relatively unchanged. This might indicate that the alterations in the Venus flytrap's metabolism depend both on the type of substance and on its amount.

## Significance Statement

It is still currently unknown how *Dionaea muscipula*—the Venus flytrap—metabolizes its prey and how the products derived from prey's digestion feed into the plant's metabolism. By feeding live crickets and purified substances to the traps, we uncovered the metabolic fate of prey components using an integrative approach based on metabolomics and transcriptomics, and found that *Dionaea* discriminates, among prey‐derived substances, those serving as nutrients from other metabolites which likely serve different roles.

## INTRODUCTION

In the last decade, the Venus flytrap (*Dionaea muscipula*, family: Droseraceae) advanced from a fancy specimen for studying plant electrical excitation to a model carnivorous plant accessible to metabolic and molecular analyses. Typical victims of *Dionaea muscipula* are small insects and arachnids that searching for food, are attracted by the vivid colors and food‐mimicking smell of the traps. When these prey arrive on the trap, they collide with the trigger hairs located on the surface, causing the trap to close. Darwin, who was actually the first to stimulate flytraps with several nutrient sources, long suspected that the active traps of *Dionaea* possessed a physical and a chemical sensory system to perceive the presence and the chemical constitution of the prey (Chase et al., 2009; Darwin & Darwin, 1888; Król et al., 2012). We now know that *Dionaea* integrates different prey‐derived information to coordinate its hunting cycle to ensure an effective cost–benefit relationship. *Dionaea* estimates prey size and nutrient content through electrical signals (action potentials, APs) elicited by the entrapped prey using a complex mechanosensory system (Böhm et al., 2016; Hedrich & Kreuzer, 2023; Hedrich & Neher, 2018; Scherzer et al., 2019). This sensory system, in addition to the mechanostimulation induced by the bending of the trigger hairs, may also involve chemosensing of some prey‐derived nutrients.

In any case, the generated APs (and associated Ca^2+^ waves) rapidly propagate through the entire capture organ (Scherzer et al., 2022; Suda et al., 2020) and are “counted”, translated into the synthesis of the touch hormone jasmonic acid (JA) (Böhm et al., 2016), and after the initial, reversible trap closure, they cause the trap to become hermetically sealed if more than 5 APs are generated. JA and its precursor, 12‐oxophytodienoic acid, then induce the secretion of the digestive fluid which completely fills the trap surrounding the prey (Escalante‐Perez et al., 2011). When prey animals are captured, the components of the exoskeleton (chitin and lipoproteins) and soon after, the internal organs of the prey, are exposed to a viscous fluid composed of mucilage, ions, and lytic enzymes secreted by the digestive glands which are present on the inner trap surfaces. The digestive fluid, in addition to chitinases, also contains several proteases. The latter completely hydrolyze the prey proteins into their amino acid building blocks (Fasbender et al., 2017; Schulze et al., 2012).

The organic nitrogen acquired from the animal foodstock is then redistributed through the trap and petiole vascular system to support the development of new traps and thereby enhance the probability of prey capture (Kruse et al., 2014). A *Dionaea* trap feeding experiment with ^13^C/^15^N‐labeled glutamine showed that the nitrogen derived from glutamine is already separated from its carbon skeleton in the decomposing fluid secreted into the green stomach formed by the closed and sealed trap. Glutamine‐derived nitrogen fueled different metabolic processes, especially amino acid (AA) metabolism, as indicated by the increased level of alanine, aspartate, valine, isoleucine, and serine. Glutamine‐derived carbon was instead respired *in loco*, as indicated by the emission of labeled CO_2_ (^13^C‐CO_2_), by the increased abundance of metabolites from the tricarboxylic acid (TCA) cycle (derived from respiratory glutamine degradation) and of proteins involved in respiratory processes (Fasbender et al., 2017). Apparently, the photosynthetic, carnivorous *D. muscipula* does not use the carbon skeletons of N‐rich amino acids as building blocks in carbon metabolism. In contrast, prey‐derived carbon skeletons serve as immediate substrates for respiratory energy production. This extra energy supports the production and secretion of the lytic enzyme cocktail providing for sustained prey decomposition and nutrient uptake. This behavior, however, is not necessarily reflected in other carnivorous plants. The tropical pitcher plant, *Nepenthes insignis*, has been shown to incorporate the carbons derived from feeding on labeled alanine into the synthesis of a defensive naphtoquinone, plumbagin (Rischer et al., 2002). Also, in a more recent analysis involving four species of carnivorous plants from distant lineages, it was shown that carbons from labeled prey were clearly assimilated by the traps, although it remains to be determined how and where insect‐derived carbons are incorporated into the plant's metabolism (Lin et al., 2025).

On the molecular side, the metabolic processes of secretion, prey digestion, and absorption are accompanied by pronounced trap transcriptomic rearrangements. Canonical stress‐related pathways already known from non‐carnivorous plants after, for example, pathogen attack, are activated, including JA biosynthesis and signaling, as well as ROS signaling, thus adding further support to the hypothesis that carnivory very likely evolved from defense mechanisms (Bemm et al., 2016). When comparing the gene expression profiles across different tissues of *D. muscipula*, the group of Bemm et al. identified a group of around 50 hydrolases specifically expressed in secretory glands (Bemm et al., 2016). A major fraction of these was identified as components of the lytic enzyme cocktail, including different proteases and chitinase types. Proteomic analysis of the digestive fluid could confirm active secretion of these hydrolases upon stimulation by touch, coronatine, or feeding (Schulze et al., 2012).

Also, when the genome of the Venus flytrap was sequenced and compared with its close members within the Droseraceae family (*Aldrovanda vesiculosa* and *Drosera spatulata*) (Palfalvi et al., 2020), it emerged that these carnivory‐specific genomes very likely evolved after a whole genome duplication and subsequent massive gene loss. In addition, species‐specific processes further shaped the plant' genomes: in *Dionaea*, formerly root‐specific genes were recruited to the trap, which is no surprise since this organ has taken over the function of nutrient uptake. The analysis of the genomes of these plants allowed the identification of the most important events associated with the emergence of carnivory. Some specific gene families were affected by a massive loss of gene copies (e.g., genes encoding regulators of root development underwent contraction, which might explain the vestigial root system of adult carnivorous plants), while other gene families specifically expanded with respect to non‐carnivorous Angiosperms. These were mainly related to all steps of prey capture and digestion. Genes involved in the attraction of insects (emission of volatile terpenoids), signal transduction, as well as those encoding various types of digestive enzymes and membrane transporters were all affected by a general increase in their copy numbers when compared with the genomes of non‐carnivorous plants. The comparison of genomes from the carnivorous Droseraceae with those of Nepenthaceae, which attract and kill insects using passive pitcher traps, showed that most of the genetic repertoire for carnivory likely evolved independently after the split of these two families (Hedrich & Fukushima, 2021; Palfalvi et al., 2020).

To gain deeper insights into the modification of trap and petiole metabolism upon digestion and adsorption of specific molecules, we fed traps with live crickets, and in parallel, with different purified compounds or macromolecules representing sources of carbon, nitrogen, and phosphorus (see below). We reasoned that the analysis of metabolic profiles following the digestion of living prey and its comparison with the feeding of single components could allow us to follow the probable metabolic fate of specific prey components by looking at the steady‐state levels of primary metabolites in both traps and petioles. We knew already that *Dionaea* could use the C and N originating from prey‐derived amino acids (Fasbender et al., 2017), using in particular amino acids as respiratory substrates (in a manner reminiscent to that observed in plant leaves following extended darkness (Araujo et al., 2011)), but little was known instead about the fate of other prey‐derived molecules.

Here, we thus ask the question of how the Venus flytrap metabolism, at the level of its traps and petioles (the part of the leaf supporting the trap), is modified upon the capture of its animal prey. To answer this question, flytraps were either fed with live crickets or well‐defined N‐ and P‐rich compounds such as casein, chitin, urea, DNA, and phospholipids. Comparing the metabolite profiles of organically fed traps with JA‐stimulated empty ones, we revealed which amino acids were invested for lytic enzyme production and which were gained from catabolism of the metabolic components of the prey.

## RESULTS AND DISCUSSION

### Assessing the metabolic code of nutrient usage upon prey capture

**Figure 1:**
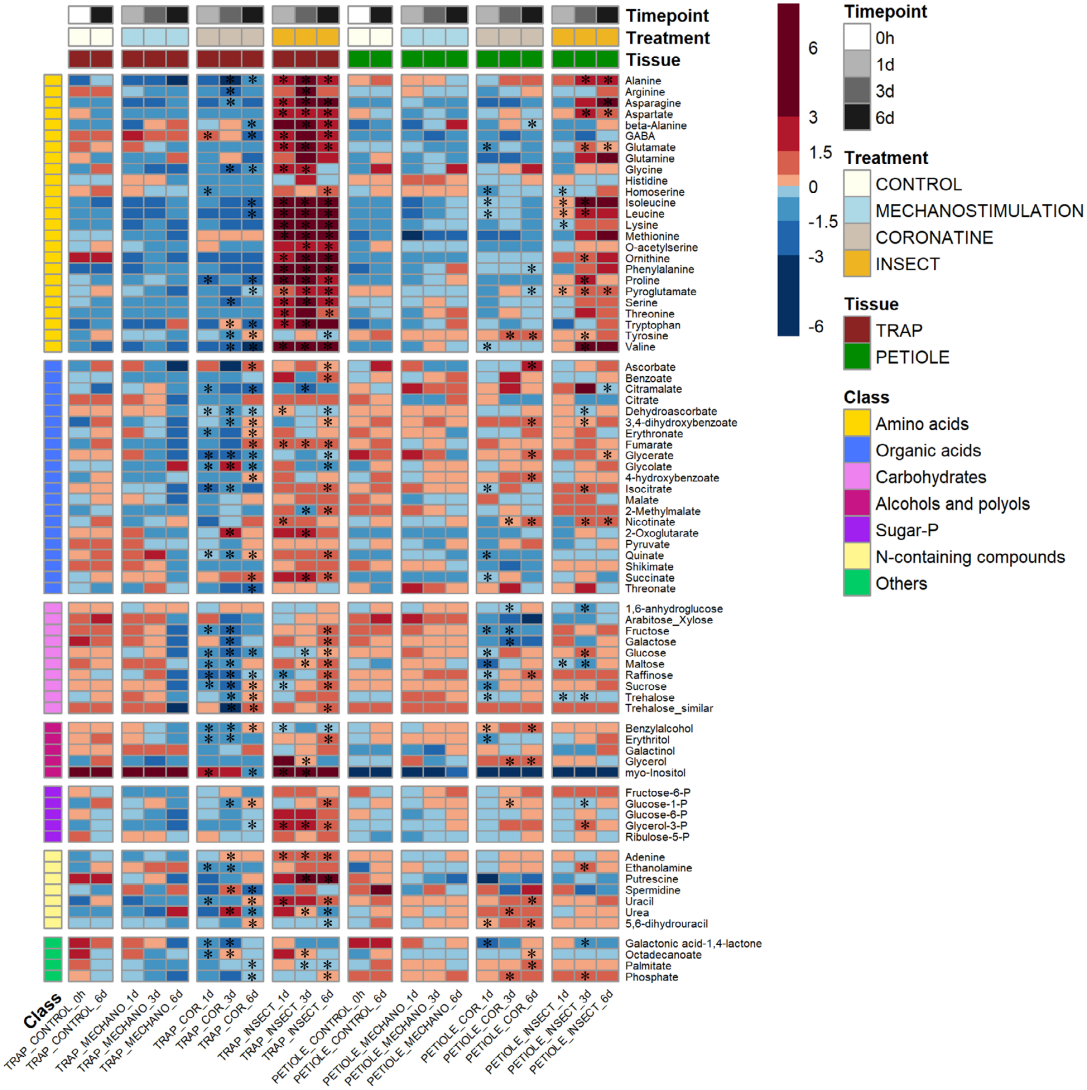
Changes in metabolite abundance in traps and petioles in response to mechanical stimulation, coronatine treatment, and insect feeding of traps. Traps were either mechanically stimulated (by gently moving inside the trap a wet filter paper strip soaked in water), sprayed with 100 μm coronatine, or fed with insects and harvested after Days 1, 3, or Day 6. Traps (and associated petioles) that were left completely untreated are labeled as “control” and were sampled only at 0 h and Day 6. The heatmap represents the log2‐transformed and mean‐centered normalized metabolite intensities, so that metabolites in red have their mean intensities greater than the metabolite mean across all conditions (and the opposite for metabolites in blue). Asterisks denote whether the change in absolute intensities (i.e., non‐log2 transformed and non‐mean‐centered) of the coronatine and insect‐treated samples is significant with respect to the related trap/petiole mechanostimulation samples at the respective timepoint (Wilcoxon pairwise test, adj *P*‐value <0.05).

To answer the question of how *Dionaea* metabolizes its prey, traps were first fed with an insect meal in the form of live crickets (*Acheta domesticus*, family Gryllidae). One, three, and 6 days after trap closure, we separately harvested the traps and associated petioles to assess the profiles of their primary metabolites with gas chromatography time‐of‐flight mass spectrometry (GC‐ToF‐MS), a much faster profiling approach with respect to single quadrupole‐MS, which is able to cover amino acids, carbohydrates, sugar‐alcohols, organic acids, and other small polar compounds (Figure 1, Figure S1 and Table S1). As a control, we supplied traps with wet filter paper strips soaked in water, which, upon application, were gently moved inside the trap (a condition we denoted as “mechanostimulation” in Figure 1). Upon capture, the movement of crickets, which often stay alive inside the trap for 1–2 days and continue to stimulate the trigger hairs, provokes long‐term trap electrical activity, JA biosynthesis, and finally lytic enzyme production and fluid phase secretion. This means that data derived from the trap's metabolome represent the sum of prey‐derived substrate inflow, secretion‐associated substrate outflow, and cellular metabolites of the tissues of the trap itself. To quantify the metabolic cost of fluid secretion itself, we stimulated traps, in the absence of prey, by spray application of 100 μm coronatine (COR), which, as an analog of JA, mimics its effects, that is, activating the secretion of digestive fluid.

Following insect feeding, pronounced metabolic rearrangements took place already after 24 h. The most striking result is the over‐accumulation in the traps of almost all AAs and of some TCA cycle intermediates over time (i.e., succinate, and 2‐oxoglutarate Figure 1 and Figure S1). These increases are accompanied by an accumulation of glucose‐1‐P (at 3 and 6 days) and of glycerol‐3‐P (at Days 1, 3, and 6), indicating a general activation of glycolysis to fuel respiration (Figure 1 and Figure S1). In coronatine‐stimulated traps, however, a drop in many AAs was observed, indicating that the relative increases of most of the metabolites detected in insect‐fed traps derive from the digestion of the insect itself. Some of the AAs over‐accumulating in the insect‐fed traps later also increased in the petioles, an indication of possible transport processes between these two organs. The large increase of methionine in insect‐fed traps points to the activation of trap sulfur metabolism, a prerequisite to sustain the production of the sulfur‐rich lytic enzymes present in the digestive fluid (Fasbender et al., 2017; Schulze et al., 2012).

Clearly, assessing the precise metabolic origin of the increases detected in the traps and petioles following prey feeding will need further experimentation, by means of feeding the plant, for example, an insect reared on a labeled substrate. Given, however, that we did not observe the same increases (of the same magnitude) in the coronatine‐treated samples (in which secretion is induced), we are inclined to assign the differential metabolite increases (= increases following the prey's digestion minus increases following the coronatine treatment) as those that can be reasonably derived from the prey.

**Figure 2:**
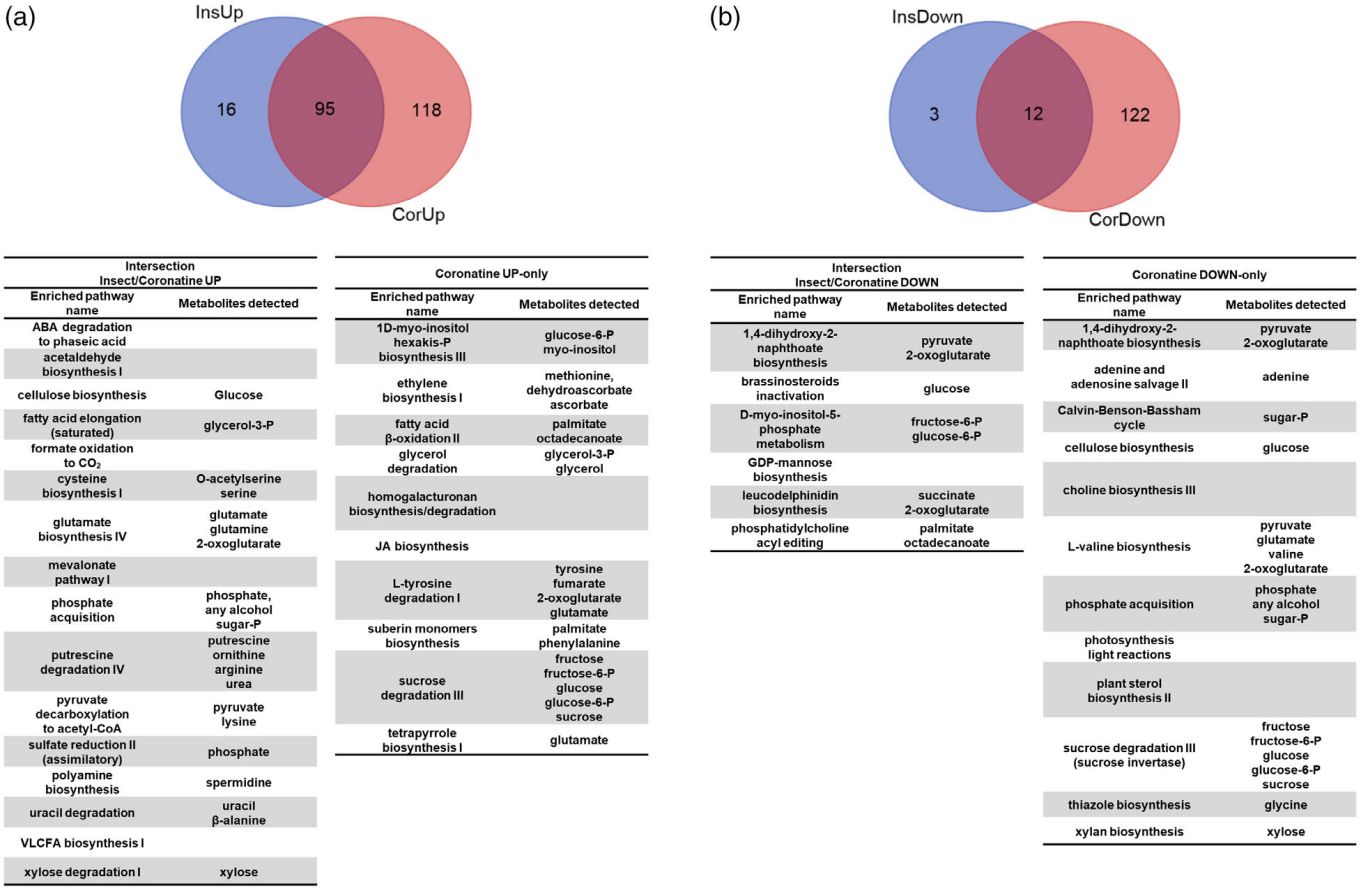
Pathway enrichment analysis of the DE metabolic genes in the different sectors of the Venn diagrams. (a) Venn diagram representing the upregulated genes upon the insect meal (“InsUp”) and the coronatine treatment (“CorUp”). (b) Venn diagram representing the downregulated genes upon the insect meal (“InsDown”) and the coronatine treatment (“CorDown”). The enriched (i.e., overrepresented) pathways in each sector, along with the respective detected metabolites, are indicated below the diagrams. No pathways were statistically overrepresented in the DE genes from the “InsUP” and “InsDown” sets. See text and Table S2 for details.

To correlate the metabolomic data described above with our previously collected transcriptomic data (Bemm et al., 2016), we first selected the differentially expressed genes (DEGs) that were in association with metabolism from the total number of DEGs after insect or coronatine stimulation (see Experimental procedures section) and then visualized the intersection of these two DEG datasets (Figure 2 and Table S2).

The upregulated metabolic DEGs in the intersection (that is, those shared by the insect meal and by treatment with coronatine) indicate a typical plant defense reaction that in the carnivorous plant is rewired for offense: *Dionaea* is activating its exocrine system (chitinases, proteases, phosphatases) to digest its prey. Furthermore, the trap takes over root‐like functions, such as nutrient mobilization, transport, and absorption. The enriched (overrepresented) pathways in the intersection in fact represent the typical pathways of nutrient assimilation (sulfur, phosphate) or are generally associated with the activation of biosynthetic capacities (biosynthesis of cysteine, glutamine, polyamine, and very long chain fatty acids). Some of these pathways are those typically active in response to conditions of flooding– and subsequent hypoxia– for example, acetaldehyde biosynthesis (Kimmerer & Macdonald, 1987), ABA degradation to phaseic acid (Gonzalez‐Guzman et al., 2021), and formate oxidation to CO_2_ (Andreadeli et al., 2009; Hourton‐Cabassa et al., 1998). The external stomach of *Dionaea* is filled with mucilage and digestive fluids secreted by glands, and as such, may constitute a low oxygen containing compartment. This is consistent with the digestive traps of other plant genera, that is, the Lentibulariaceae *Utricularia* and *Genlisea*, which were shown to operate under almost anoxic conditions leading to the hypothesis that prey may actually be killed by suffocation (Adamec, 2007). Indeed, two other overrepresented pathways in the intersection of upregulated genes between insect and the coronatine treatment are those of saturated fatty acid elongation and synthesis of very long‐chain fatty acids (VLCFAs). Transcription of lipid metabolism genes and the amount of several lipid classes themselves are heavily affected by anoxia, and the increase usually observed in VLC ceramides under anoxia, in particular, is known to confer tolerance to plant organs upon submergence (Xie et al., 2021).

On the other hand, few metabolic genes (16) are upregulated upon the insect meal only, and despite the absence of specific overrepresented metabolic pathways, a specific isoform of chitinase (comp168951_c1, log2 fold‐change 9.02, adj pval 3.74E‐30) was detected. This chitinase is part of a larger group of chitinases which are otherwise upregulated also in response to coronatine treatment. This may imply that at least some isoforms are, directly or indirectly, responsive to the physical presence of chitin within the trap. Other genes exclusively upregulated upon the insect meal include two isoforms of beta‐amylase, presumably involved in starch degradation (a process necessary to release carbon reserves to support digestion of the insect, given that starch is absent in the prey), and the transcript encoding the inositol polyphosphate phosphatidylinositol 5‐phosphatase (comp231562_c0, log2 fold‐change 8.37, adj pval 2.86E‐68), a gene involved in the coordination of several processes associated with endocytosis (Golani et al., 2013).

### The capture of prey and the metabolic cost of secretion of the digestive fluid

In addition to the increase of AAs and other metabolites likely derived from the prey, there are also several changes that occur upon coronatine treatment alone and are thus irrespective of the presence of the prey. In the traps, these changes involve most of the carbohydrates, some AAs, and several organic acids (e.g., erythronate, glycerate, glycolate, isocitrate, threonate, see Figure 1 Table S1 and Figure S3). Generally, carbohydrates decrease in coronatine‐treated traps (with respect to mechanostimulated traps) with more specifically fructose, glucose, maltose, raffinose, and sucrose showing a decrease at Days 1 and 3, with a subsequent increase limited to glucose, sucrose, and trehalose at 6 days after coronatine treatment (Figure 1, Table S1 and Figure S3). An increase at Day 6, following coronatine treatment and in comparison to mechanostimulated traps, is also observed in TCA cycle intermediates (fumarate, malate, succinate, erythronate and 4‐hydroxybenzoate, Figure 1, Figures S1 and S3). This may indicate that traps, once they enter into “secretion mode” (which is activated also by the coronatine treatment alone) use available carbohydrates to support energy production from the upregulated operation of the TCA cycle, at least until 3 days. After that at Day 6, carbohydrates increase, perhaps following starch degradation to further support the TCA cycle. Several AAs including β‐alanine, GABA, proline, valine, leucine, isoleucine, and tryptophan show instead a decrease in coronatine‐treated traps with respect to mechanostimulated controls, with no final recovery at Day 6 (Figures S1 and S3), an indication that they probably feed the TCA cycle through those catabolic reactions typical under conditions of high energy demand (Araujo et al., 2011; Hildebrandt et al., 2015).

Comparing our transcriptomic data with the metabolic profiles of stimulated *Dionaea*, we can conclude that although we have a relatively large overlap of transcriptional induction of “metabolism‐related” genes both after coronatine and insect feeding, the two conditions actually have rather opposite effects on trap metabolism. The capture of the insect greatly affects the amount of primary metabolites, given that most of the increases, especially those at Day 1 and Day 3, likely represent the influx of components coming from the digestion of the prey. Coronatine treatment, on the other hand, rather induces changes of the trap/petiole endogenous metabolism related to the secretion of the digestive fluid itself. In this latter case, most of the metabolites decrease at Day 1 and Day 3, with a late recovery only at Day 6 (at least for carbohydrates and for some TCA cycle intermediates). In the absence of prey, the changes of primary metabolites occurring in the presence of coronatine thus represent the “net metabolic cost” of secretion of the digestive fluid. Upon artificial stimulation of the traps (either by coronatine or by magnet‐based stimulation) in fact, an increase in the production of digestive enzymes has been observed (Schulze et al., 2012).

After insect capture, the traps immediately close and the series of action potentials elicited by the moving prey induces the accumulation of the “touch” hormones 12‐oxophytodienoic acid (12‐OPDA) and JA. These molecules transduce the signal initiating secretion of the digestive fluid by the trap glands (Libiakova et al., 2014). (Bemm et al., 2016; Escalante‐Perez et al., 2011) To check whether the insect prey constitutes a specific replenishment for the AA investment necessary for the production of the digestive enzymes (in a single trap), we surveyed the AA composition of the most abundant protein of the digestive fluid, dionain‐1 (Schulze et al., 2012; Takahashi et al., 2011), a cysteine proteinase (Genbank ID: AKA58504) representing >60% of all proteins normally present in the digestive fluid upon insect or coronatine stimulation. As in all other related cysteine proteinases, dionain‐1 is particularly rich in Ala, Gly, and Ser, with these three AAs constituting almost 30% of the whole sequence. A food source able to specifically replenish the plant's AA investment in the production of these proteins should thus be rich in these small amino acids and should be present in a sufficient amount to compensate for the amino acid investment for the production of the digestive fluid in a single trap (at its maximum, i.e., 4 days after stimulation, the total protein content in the digestive fluid is around 4 mg per trap, see Scala et al., 1969 and experimental procedures). Crickets (*Acheta domesticus*) represent protein‐rich sources (reaching over 40% of protein content (Montowska et al., 2019)); as such, even for a small cricket having a mass of around 0.5 g and considering a protein digestibility of around 84% (Oonincx & Finke, 2020), the amino acid intake from the complete digestion of the prey largely exceeds the pool of amino acids needed to produce the digestive enzymes.

### Phloem‐based trap‐petiole metabolic interconnection is selective

**Figure 3:**
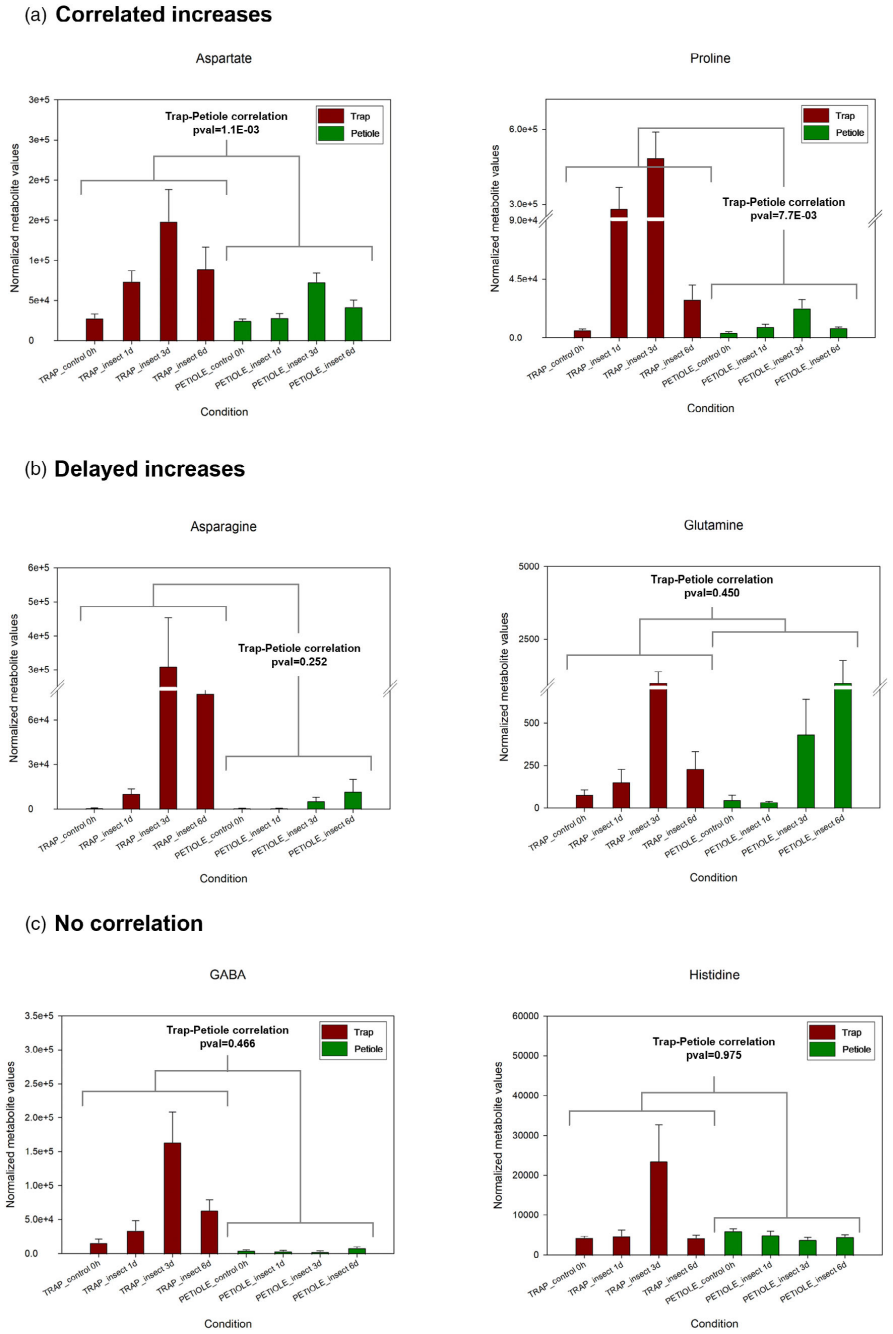
Transport processes between trap and petiole affect the content of some amino acids. Following insect capture and digestion, the variation in the content of amino acids in traps and petioles follows three different tendencies. In the case of aspartate and proline (a), the increases detected in the trap are accompanied by correlated, simultaneous increases in the petiole. In the case of asparagine and glutamine (b), although in both traps and petioles its content increases, this tendency is not exactly reflected in the petiole, where the detected increase is delayed, showing a peak only at Day 6. In the case of GABA and histidine (c), the increases detected in the trap are instead not reflected in the petiole. Data are expressed as average normalized metabolite values (a.u.) ±SE. The *P*‐values from the repeated measures correlation between trap and petiole are indicated in each histogram.

Following insect stimulation, some of the metabolic changes observed in the traps were also reflected, with different magnitudes, in the petiole. In general, amino acids, likely deriving from the prey, and whose content was initially less abundant in the petiole with respect to the trap, after some days increased in the petiole as well. We recognized three different tendencies in the variation of amino acid content between the insect‐fed trap and the adjacent petiole. In the case of aspartate, proline, leucine, lysine, and threonine, which were initially almost unchanged in the petiole at Day 1, they then increased in the petiole, reaching a peak at Day 3, thus mirroring the trend observed in the fed trap (Figure 3a for asparate and proline, for other amino acids see Figure S1). In the second case, the rise of other amino acids, such as asparagine, glutamine, and isoleucine, appeared somewhat delayed in the petiole, where their abundance reached a maximum only at Day 6 (Figure 3b for asparagine and glutamine, for other amino acids see Figure S1). In the case of GABA and histidine, their increase in the insect‐fed traps was instead not reflected in the petiole (Figure 3c). In general, both the correlated and the delayed increases we observed for amino acids between the fed trap and the adjacent petiole may indicate the transport of these metabolites from the “active” trap source to the “inactive” sink tissues of the petiole (Tegeder & Hammes, 2018). This transport shows apparently two different temporal dynamics. The first is the one affecting aspartate and proline (and branched‐chain amino acids), where their abundance in the petiole mirrors the one observed in the trap. Other amino acids, on the other hand, increase first in the trap (peaking at Day 3), and only after a delay, they increase in the petiole as well (with a maximum at Day 6). This delayed increase is indicative of a phloem‐based translocation, mediated by active transporters, affecting only certain amino acids (Tegeder & Hammes, 2018). This is particularly evident for asparagine and glutamine, the two most abundant amino acids in the phloem sap of several plant species (Broussard et al., 2023; Tegeder & Masclaux‐Daubresse, 2018). These two transport patterns are common to several amino acids but are instead not observed in other primary metabolites. For example, the increases of isocitrate, succinate, glycerol‐3‐P, and putrescine, which were all detected in the traps following insect feeding, were not reflected in the petioles, where the content of these metabolites remained unchanged with respect to the petiole water‐ and coronatine controls (Figure S1). Most of the organic acids do not show a significant increase in the trap between Days 1 and 3 following insect capture, and the same can be said for most carbohydrates, including glucose, fructose, and galactose (Figure S1). Although it is not possible to *ad hoc* exclude a basal level of transport of these metabolites to the petiole, the lack of correlation between the increases in the two organs might indicate that these compounds are probably metabolized *in loco* (in the trap), a modality consistent with the high energy demand required for the secretion of the digestive fluid and the subsequent absorption processes (Fasbender et al., 2017).

### Does a single component of the prey endogenous pool cause a shift in the metabolic profile of trap or petiole?

To gain deeper insights into the modification of trap and petiole metabolism upon digestion and adsorption of specific molecules, we pre‐stimulated traps with 100 μm coronatine for 12 h to induce initial secretion. We then fed the traps with chitin, casein, urea, nucleic acids (salmon sperm DNA) or a phospholipid mixture to harvest control and treated traps and petioles after 1, 3, and 6 days.

Coronatine stimulation alone resulted (with respect to untreated controls at 0 h) in the traps in the immediate reduction of most carbohydrates (Figure S2). This pattern may be explained by the need for fast energy consumption to sustain the process of secretion. This was accompanied by the increase of succinate starting from Day 1, which may indicate the switch from photosynthetic energy gain to respiration (Wolucka et al., 2005).

**Figure 4:**
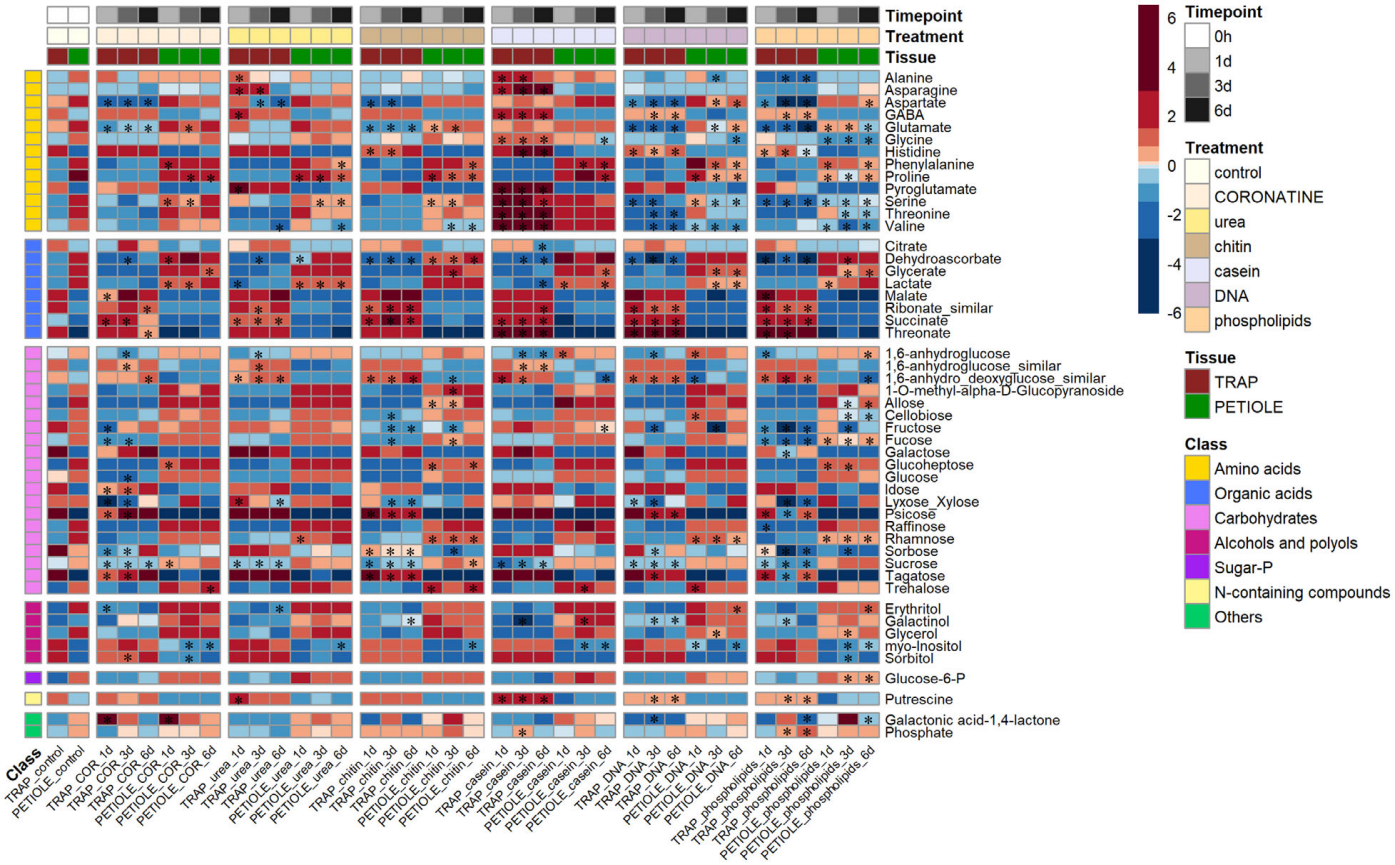
Changes in metabolite abundance after feeding secreting traps with urea, chitin, casein, DNA, or phospholipids. Traps were pretreated with 100 μm coronatine, fed with the respective substances, and harvested after 1, 3, or 6 days. The conditions “TRAP control” and “PETIOLE control” refer to plants (i.e., traps and petioles) sampled after 12 h from the application of coronatine (no feeding). The heatmap represents the log2‐transformed and mean‐centered normalized metabolite intensities, so that metabolites in red have their mean intensities greater than the metabolite mean across all conditions (and the opposite for metabolites in blue). Asterisks denote whether the change in absolute intensities (i.e., non‐log2 transformed and non‐mean‐centered) is significant with respect to the “TRAP control” for trap samples or to the “PETIOLE control” for petiole samples (Wilcoxon pairwise test, adj *P*‐value <0.05).

Additional casein feeding led to an increased abundance of all amino acids in the traps (Figure 4 and Figure S2). The increase of amino acids in casein‐fed traps, with respect to the coronatine controls, may directly contribute to respiration and energy supply. Basically, all amino acids can be converted into precursors or intermediates of the TCA cycle, either directly (e.g., asparagine, aspartate, glutamine, glutamate) or through pyruvate/Acetyl‐CoA (Hildebrandt et al., 2015). The increase in pyroglutamic acid, a non‐proteinogenic amino acid, is instead probably related to the extent of protein (casein) digestion. Pyroglutamic acid derives, in fact, from the post‐translational modification, at acidic pH, of a peptide/protein N‐terminal glutamine or glutamate.

Based on the massive upregulation of chitinase genes in secreting traps, even in the absence of prey (see Table S2), we next decided to directly feed chitin after pre‐stimulation of the traps with coronatine. From previous data, we knew that genes encoding chitinases were already over‐expressed following simple mechanostimulation or chitin feeding (Bemm et al., 2016). From the metabolic point of view, however, chitin feeding did not result in drastic metabolite changes. Particularly, amino acids, and in contrast to what was observed upon casein feeding, do not show any increase after chitin feeding compared with coronatine‐treated traps. This may be due to two, not mutually exclusive, factors. The first is that casein is more readily digested with respect to chitin, making casein‐derived nitrogen more readily incorporated in the trap's metabolism, a result in agreement with previous data, where protein‐derived N is absorbed 4–8 times more effectively than chitin‐derived N, thus rendering protein digestion and absorption much more beneficial for the plants (Pavlovic et al., 2016). Since most of the secreted chitinases are endochitinases (including the most abundant secreted chitinase, VF‐I (Bemm et al., 2016, Paszota et al., 2014)), which cleave the internal bonds of the chitin polymers, it is possible that the resulting oligomers of GlcNAc (chitooligosaccharides) may act solely as signaling molecules in the digestive stomach, without being absorbed by the trap. This is consistent with the presence and upregulation of the LysM‐type chitin receptor DmCERK1 in insect‐feeding traps (Bemm et al., 2016) and with the known role that glucosamine‐containing oligomers have in eliciting a defense response (Wan et al., 2008). The second factor contributing to the substantial lack of (chitin‐derived) nitrogen incorporation into the primary metabolites of the trap might involve a dose–response mechanism that would depend on the absolute amount of supplied nitrogen. In fact, the amount of the applied nitrogen in the case of chitin feeding is around 60% of the nitrogen supplied from casein feeding (nitrogen supplied/trap: 4.5 mg for casein and 2.76 mg for chitin).

Increased supply of nitrogen by urea or DNA feeding to the secreting trapdid not largely affect the levels of primary metabolites. This might be again the consequence of very different nitrogen amounts applied in feeding the traps with urea and DNA (nitrogen supplied/trap: 90 μg for urea and 1.4 mg for DNA). Only upon urea feeding, and with respect to coronatine‐treated traps, we detected transient increases of a few amino acids, including asparagine, aspartate, pyroglutamic acid, and glutamate (these latter two only at Day 1) (Figure S2). This finding is in contrast to what is known from other carnivorous plants: the pitcher plant *Nepenthes hemsleyana* features a mutualistic relationship with the bat *Kerivoula hardwickii* and can feed on urea contained in the bat' excrements supplied to the trap. Although ureases needed to metabolize this compound are closely related in Nepenthes and Dionaea, feeding on urea appears less pronounced in the Venus flytrap (Yilamujiang et al., 2017).

The feeding with phospholipids did not cause either large alterations of primary metabolites in the traps, except for a gradual increase of phosphoric acid and threonic acid. This trend was mirrored by the feeding with DNA (a rich P‐source), an indication that both phospholipid‐ and nucleic acid‐derived P, despite the different absolute amounts of P supplied (0.81 mg for DNA and 0.04 mg for phospholipids), may actually contribute to the level of free phosphoric acid.

Thus, in general, the lack of large alterations in the level of trap metabolites following substance feeding (except perhaps for casein) may be reconducted both to the substance type and to the supplied amount of the specific substance. Although it is indeed likely that more evident alterations could be observed by applying higher amounts of N and P, it is also possible that chitin breakdown products, oligomers of DNA, and small lipid‐derived molecules may act exclusively as ligands in the trap extracellular space, interacting with specific receptors to activate a variety of responses in defense, adsorption of molecules, or to stimulate further fluid/enzyme secretion.

Regarding the metabolomic pattern of the petiole tissue, a similar picture as in the trap emerged (Figure 4 and Figure S2). With respect to the coronatine control, casein increased the amount of some amino acids (aspartate, phenylalanine and valine) at either one or more time points, a trend probably reflecting the different dynamics of transport processes occurring between the trap and the petiole. While the initial decreases in the abundance of some carbohydrates in the trap/petiole after coronatine stimulation (and with respect to the non‐stimulated traps/petioles) might reflect the energy demand required for secretion, the increase in the abundance of aspartate, phenylalanine, and valine after casein consumption also points to increased respiration. The petiole therefore supports the trap during the energy‐consuming process of secretion.

Taken together, we dissected the metabolic alterations induced by the digestion of prey from those induced by the sole secretion of the digestive fluid. One of the main findings is that secretion *per se* is an energetically costly process, where not only carbohydrates, but also amino acids are probably used as respiratory substrates and, naturally, also as building blocks of the hydrolases present in the digestive fluid. Interestingly, while transcriptomic rearrangements of metabolic genes are quite similar after insect feeding and coronatine stimulation, both treatments affect *Dionaea*'s own metabolism in opposite directions. Therefore, complementary methods are needed to study the complex interrelation between prey nutrient uptake and the flytrap's metabolism.

In this context, one should especially pay attention to the selective shuttling of nutrients or metabolic compounds between the trap and the petiole. Neither the action potentials nor the Ca^2+^ wave nor indeed—at later stages of digestion—the jasmonate signal is transmitted from trap to petiole (Pavlovic et al., 2017; Pavlovic & Mithofer, 2019; Suda et al., 2020). That means there is no electrical or hormonal interconnection between these tissues in response to prey capture. The data shown here, however, indicate that there is at least some selective metabolic coupling which also allows the trap to energetically support the petiole.

Upon feeding with different sources for N and P, only protein (casein) evoked large effects on the levels of primary metabolites, suggesting that the Venus flytrap's absorption metabolism responds both to the absolute dose of the supplied substance but may also discriminate on the basis of the type of substance to be digested and eventually incorporated. It is thus possible that chitin, nucleic acids, and phospholipids and breakdown products thereof might, rather than directly being incorporated, act as prey‐derived signaling components. Future experiments are, however, required to clarify the possible exclusive metabolic or signaling role of these substances and establish the molecular mechanisms underlying such signaling pathways.

## EXPERIMENTAL PROCEDURES

### Plant material

Wild‐type *Dionaea muscipula* plants were purchased from CRESCO Carnivora (Netherlands) and grown in plastic pots at 22°C/18°C (day/night) in a 16 h:8 h light– dark photoperiod. An automatic water spraying system was used to maintain high humidity. All experiments were performed with healthy mature plants (2–3 years old, rosette diameter 10–15 cm). For feeding, adult traps (2–2.5 cm in length) were chosen. Groups for each treatment were composed of at least six plants (six biological replicates/treatment). Non‐stimulated traps and petioles were immediately frozen in liquid nitrogen and stored at −80°C.

For insect or coronatine treatment (Figure 1), traps were stimulated by feeding traps with live crickets (*Acheta domesticus*, family Gryllidae) or by spraying a 100 μm coronatine solution (Sigma‐Aldrich, St. Louis, MO, USA) directly onto open traps. As a mechano‐stimulated control, traps were closed by supplying them with a wet filter paper strip. Traps and corresponding petioles of control or treated traps were harvested 1, 3, or 6 days after stimulation onset, immediately frozen in liquid nitrogen, and stored at −80°C until further use.

For feeding individual compounds (Figure 4), untreated control traps and corresponding petioles were harvested. Then, traps were primed by spray application of a 100 μm coronatine solution (Sigma‐Aldrich) to induce secretion and transcriptional activation of digestive enzymes and nutrient transporters. For feeding, 12 h after coronatine had been applied, the secreting traps were forced open with a forceps and supplied with urea, chitin, casein, DNA, phospholipids, or nothing (controls). Therefore, small amounts of chitin, casein, or salmon sperm DNA were moistened with distilled H_2_O, and small balls (diameter 5 mm) were formed and placed inside the opened trap. For urea or phospholipid feeding, filter paper strips soaked with 100 μl 30 mm urea or 10 mg/ml phospholipid mixture (Soy Phospholipid mixture; Avanti 690050; phosphatidylcholine (PC), phosphatidylethanolamine (PE), phosphatidylinositol (PI), phosphatidic acid (PA), and lysophosphatidylcholine (LPC) were placed inside the open trap). The absolute amount of supplied nitrogen (for each trap) was 4.5 mg for casein, 2.5 mg for chitin, 1.4 mg for DNA, and 90 μg in the case of urea. The amount of supplied phosphorus was instead 0.81 mg for DNA and 0.04 mg for phospholipids. Traps and corresponding petioles of control or treated traps were harvested 1, 3, or 6 days after feeding onset, immediately frozen in liquid nitrogen, and stored at −80°C until further use.

Generally, traps were washed thoroughly with water shortly before harvesting to remove all remainders of fed substances or prey insects.

### Metabolic profiling

Primary metabolites of *D. muscipula* traps and petioles were analyzed by GC‐ToF‐MS (gas chromatography‐time of flight‐mass spectrometry). At the indicated time points, following the various treatments described in the text (insect, coronatine stimulation and feeding of specific compounds), the harvested trap and petioles were immediately snap‐frozen in liquid N_2_ and stored at −80°C until further analysis. For each analysis, six biological replicates were taken.

Approximately, 50 mg of frozen sample material was weighed into a cooled 2 ml eppendorf tube containing 600 μl cold methanol (spiked with 20 ng/μl of β‐phenyl‐glucoside as internal standard). Tubes were vortexed briefly, heated to 65°C, and shaken at 1400 ***g*** for 15 min. Samples were then centrifuged at 10 600 ***g*** for 10 min; the supernatant was then mixed with 300 μl of chloroform and 600 μl of water. After a further centrifugation at 10 600 ***g*** for 10 min, aliquots of 150 μl from the upper (polar) phase were dried in the speedvac for at least 3 h.

For derivatization, 60 μl of a 30 mg/ml^−1^ solution of methoxyamine hydrochloride in anhydrous pyridine (Sigma‐Aldrich Inc., Steinheim, Germany) was added to the dried extracts, and samples were incubated at 37°C for 2 h with shaking at 1400 ***g***. Thereafter, 120 μl of *N*‐methyl‐*N*‐(trimethylsilyl)‐trifluoroacetamide (MSTFA; containing a mixture of fatty acid methyl ester standards for correction of retention time) was added, and samples were incubated at 37°C for 30 min with shaking at 1400 ***g***. Samples were then analyzed on a GC–MS system, composed of an Agilent GC 6890 N gas chromatograph coupled to a Pegasus III time‐of‐flight mass spectrometer (Leco Instruments, St. Joseph, MI, USA), equipped with an autosampler (CTC Combi PAL; CTC Analytics, Zwingen, Switzerland). All devices were controlled by the Leco ChromaTof software (Leco Instruments).

Aliquots of 1 μl of the derivatized sample were injected in splitless mode into the system and separated on a capillary column (MDN‐35 capillary column, 30 m length). Run conditions as well as MS settings were as described by (Lisec et al., 2006). Analysis of raw data, including peak detection, peak alignment, and identification of compounds based on matching with the Golm Metabolome Database (Kopka et al., 2005), was performed with Tagfinder software (Luedemann et al., 2008). Peak areas were normalized (standardized) using the peak area of the internal standard, β‐phenyl‐glucoside, and the dry weight of samples. Missing data (i.e., metabolites not detected in some conditions/time points) were imputed through the random assignment of a value included in the interval between zero and half of the minimum detected value for that specific metabolite across all conditions (Chilimoniuk et al., 2024). A non‐parametric Kruskal–Wallis rank test followed by a Wilcoxon rank sum test (with a false discovery rate adjustment for multiple comparisons) (Hollander et al., 2015) was applied using a custom R script on the normalized peak areas to assess significant differences among the various treatments (insect/coronatine or the different feeding sources) and time points (Days 1, 3, and 6). Only for the purpose of data visualization in Figure 1 (heatmap of the insect feeding experiment) and Figure 4 (heatmap of the feeding experiment with urea, chitin, casein, DNA and phospholipids) the normalized peak areas first log2‐transformed and subsequently mean‐centered. Log transformation and centering are common data transformation techniques to reduce heteroscedasticity and remove the offset from metabolomics data (van den Berg et al., 2006). Following the insect's capture, to assess the significance of correlation between the metabolite amounts in traps and petioles (Figure 3), we calculated the *P*‐values using the method of repeated measures correlation (using the R package rmcorr). This method does not imply aggregation of the individual replicated values, with the result of achieving a higher statistical power with respect to standard correlation/regression in estimating the correlation in the presence of multiple replicates (Bakdash & Marusich, 2017).

### Venn diagrams and enrichment analysis of DE metabolic genes

Transcriptome data were based on the datasets reported in (Bemm et al., 2016), which included a feeding experiment of *D. muscipula* traps with live crickets in comparison to coronatine‐stimulated traps and petioles. To retrieve the Arabidopsis orthologs for *Dionaea* genes, we selected the conditional reciprocal best blast hit (CRB‐Blast; Aubry et al., 2014) from each ortholog initially detected in an Orthofinder run (Emms & Kelly, 2019) containing the full proteomes of *D. muscipula*, *A. thaliana*, and 13 other eudicot and monocot species. From the list of Arabidopsis orthologs corresponding to *Dionaea* DE genes (defined as those with adj *P*‐value <0.05 and fold‐change >2 or <0.5), to extract a list of DE metabolic genes, we then extracted only those contained in the Aracyc database (www.plantcyc.org), which includes a curated list of enzymes involved in metabolic pathways of primary and secondary metabolism (Zhang et al., 2005). Enrichment analysis of metabolic pathways in DE gene sets was calculated on each sector of the Venn diagrams, using a Fisher's exact test on a 2 × 2 contingency table (or a corrected Chi‐square test using continuity correction if a cell value was <5), comparing the number of DE genes belonging to a particular metabolic pathway with respect to DE/non‐DE genes belonging to the other pathways. A metabolic pathway was considered overrepresented if its *P*‐value was <0.05. To associate metabolites to specific pathways (Figure 2), we cross‐checked the substrates and products assigned to each individual pathway in Plantcyc with the metabolites we detected in our profiling experiments.

## AUTHOR CONTRIBUTIONS

Conceptualization: IK, FS, ARF, RH. Methodology: FS, TT. Investigation: IK, FS, TT. Visualization: IK, FS. Supervision: ARF, RH. Writing—original draft: IK, FS. Writing—review & editing: IK, FS, ARF, RH.

## CONFLICT OF INTEREST

Authors declare that they have no competing interests.

## Supporting information


**Figure S1.** Histograms and associated matrixes of *P*‐values for the metabolites detected in the insect feeding experiment. Data are expressed as average normalized metabolite values (a.u.) ±SE. The triangular matrixes below each histogram represent the significance of the *P*‐values for all pairwise combinations. An asterisk denotes a significant difference between two conditions (adj *P*‐value <0.05), while red circles denote a non‐significant difference (with darker reds for comparisons approaching a *P*‐value of 1). The conditions denoted as “TRAP/PETIOLE_H2O_1/3/6d” refer to mechanostimulated (water‐treated) traps and associated petioles.
**Figure S2.** Histograms and associated matrixes of *P*‐values for the metabolites detected in the substance feeding experiment. Data are expressed as average normalized metabolite values (a.u.) ±SE. The triangular matrixes below each histogram represent the significance of the *P*‐values for all pairwise combinations. An asterisk denotes a significant difference between two conditions (adj *P*‐value <0.05), while red circles denote a non‐significant difference (with darker reds for comparisons approaching a *P*‐value of 1).
**Figure S3.** Changes in metabolite abundance in trap and petiole after inducing trap secretion by spray application of 100 μm coronatine. The heatmap represents the log_2_‐fold changes of normalized metabolite abundances in coronatine‐treated samples with respect to the mechanostimulated traps and associated petioles at the respective timepoints. Asterisks denote a significant difference between the coronatine‐treated sample with respect to its mechanostimulated trap or associated petiole at the respective timepoint (adj *P*‐value <0.05, following the initial Kruskal–Wallis test and the Wilcoxon rank sum test for multiple comparisons).

**Table S1.** Dataset for insect feeding experiment. Table containing metabolite levels measured in insect‐treated and COR‐treated traps and petioles at different timepoints after feeding (1, 3, and 6 days). Values represent normalized metabolite levels (by internal standard and weight), in arbitrary units.

**Table S2.** Venn diagrams and enrichment analysis. Excel workbook containing the various steps for construction of Venn diagrams of DE genes and the identification of enriched metabolic pathways for each sector of the Venn diagrams. Enriched pathways were identified using a Chi‐square test on a 2 × 2 contingency table comparing the number of DE genes in a specific metabolic pathway with respect to the DE/non‐DE genes detected in all other metabolic pathways.

**Table S3.** Dataset for substance feeding experiment. Table containing metabolite levels measured in traps and petioles following COR treatments and after feeding with urea, chitin, casein, DNA, and phospholipids, at different timepoints after feeding (1, 3, and 6 days). Values represent normalized metabolite levels (by internal standard and weight), in arbitrary units.

## Data Availability

All data are available in the main text or in the Supporting Information.
