# Supplementary material for: Venus flytraps' metabolome analysis discloses the metabolic fate of prey animal foodstock
Source: Plant J. 2025 Aug 4;123(3):e70391. doi: 10.1111/tpj.70391 (PMC12321085; doi:10.1111/tpj.70391)
Supplement: Supplementary file 1 — Figure S1. Histograms and associated matrixes of P‐values for the metabolites detected in the insect feeding experiment. Data are expressed as average normalized metabolite values (a.u.) ±SE. The triangular matrixes below each histogram represent the significance of the P‐values for all pairwise combinations. An asterisk denotes a significant difference between two conditions (adj P‐value <0.05), while red circles denote a non‐significant difference (with darker reds for comparisons approaching a P‐value of 1). The conditions denoted as “TRAP/PETIOLE_H2O_1/3/6d” refer to mechanostimulated (water‐treated) traps and associated petioles. Figure S2. Histograms and associated matrixes of P‐values for the metabolites detected in the substance feeding experiment. Data are expressed as average normalized metabolite values (a.u.) ±SE. The triangular matrixes below each histogram represent the significance of the P‐values for all pairwise combinations. An asterisk denotes a significant difference between two conditions (adj P‐value <0.05), while red circles denote a non‐significant difference (with darker reds for comparisons approaching a P‐value of 1). Figure S3. Changes in metabolite abundance in trap and petiole after inducing trap secretion by spray application of 100 μm coronatine. The heatmap represents the log2‐fold changes of normalized metabolite abundances in coronatine‐treated samples with respect to the mechanostimulated traps and associated petioles at the respective timepoints. Asterisks denote a significant difference between the coronatine‐treated sample with respect to its mechanostimulated trap or associated petiole at the respective timepoint (adj P‐value <0.05, following the initial Kruskal–Wallis test and the Wilcoxon rank sum test for multiple comparisons). [file TPJ-123-0-s001.docx]

Supplementary Information for

Venus flytraps’ metabolome analysis discloses the metabolic fate of prey animal foodstock

Ines Kreuzer, Federico Scossa *et al.*

*Corresponding authors. Email: federico.scossa@crea.gov.it, rainer.hedrich@suat-sz.edu.cn

**This PDF file includes:**

Supporting Information Figs. S1 to S3

Legends for Supporting Information Tables S1 to S3

**Other Supplementary Materials for this manuscript include the following:**

Supporting Information Tables S1 to S3: separate Excel files


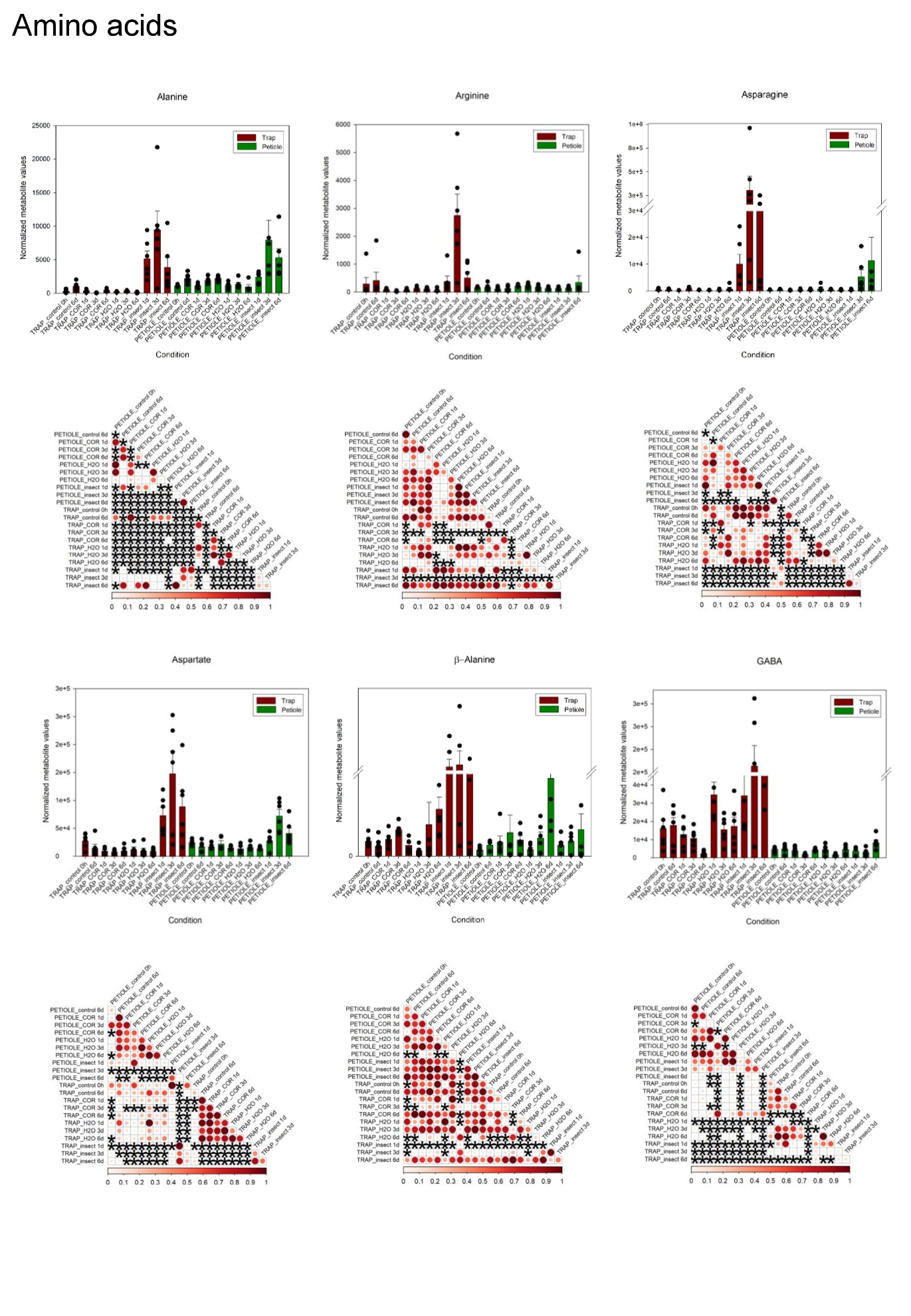

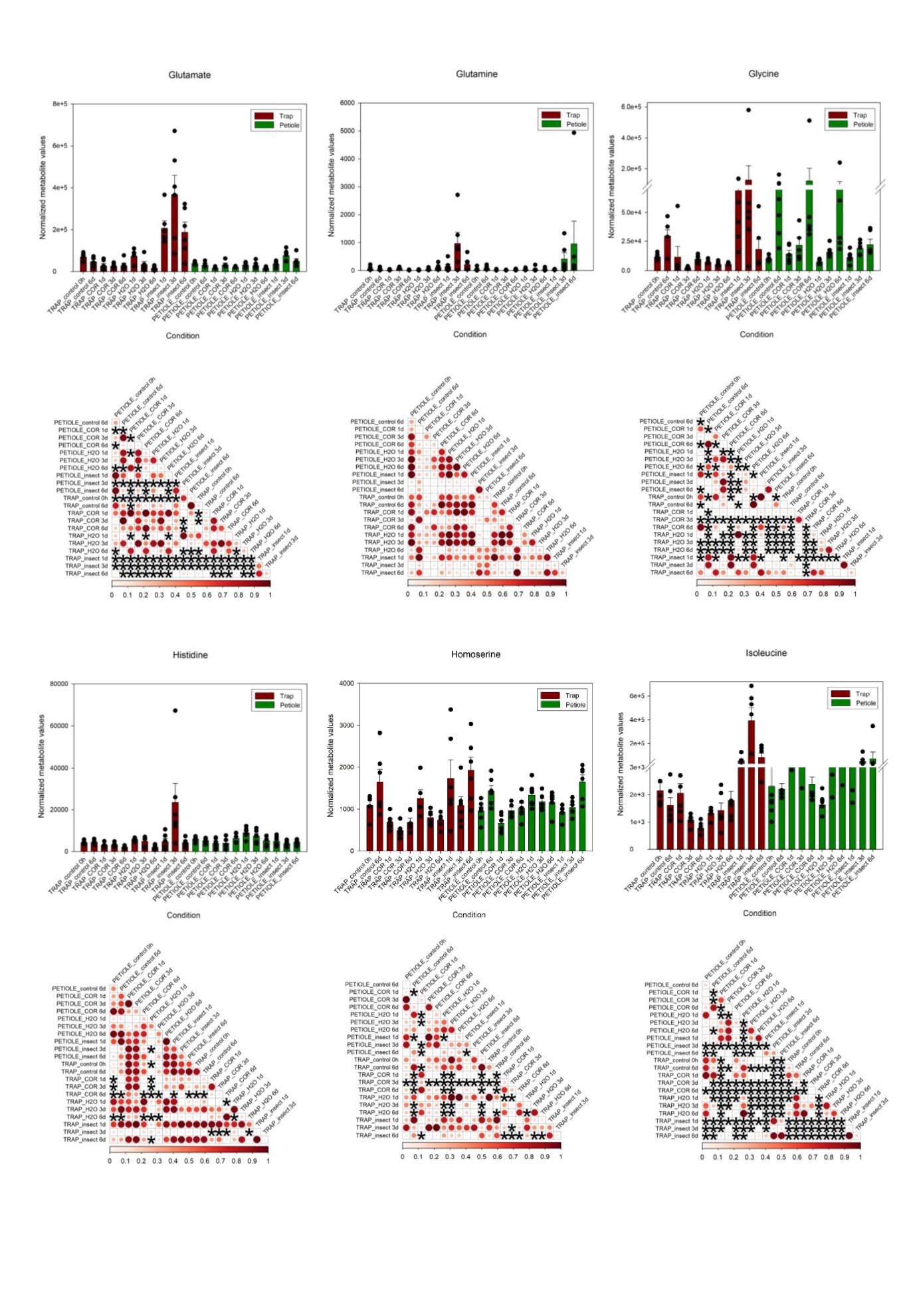

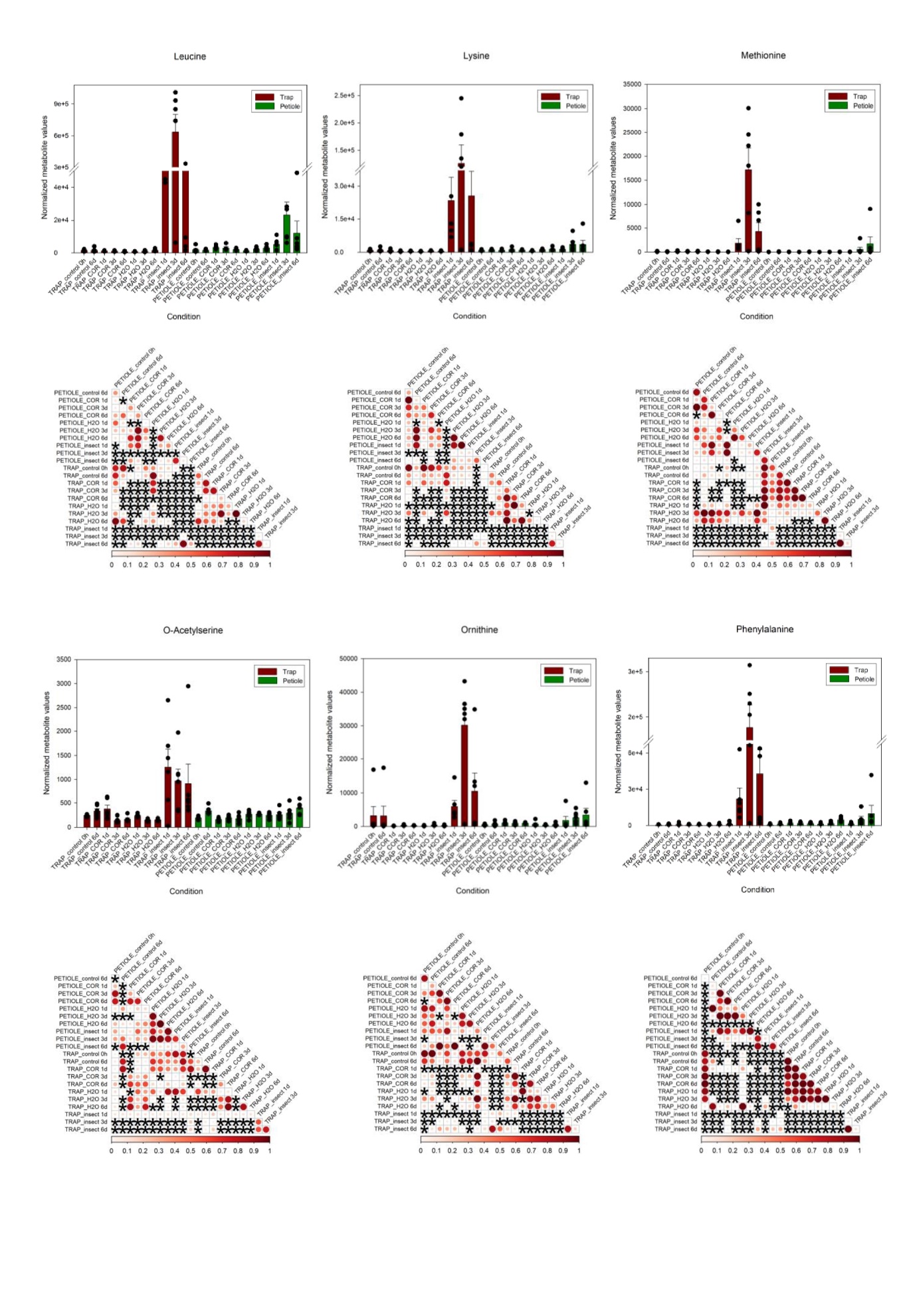

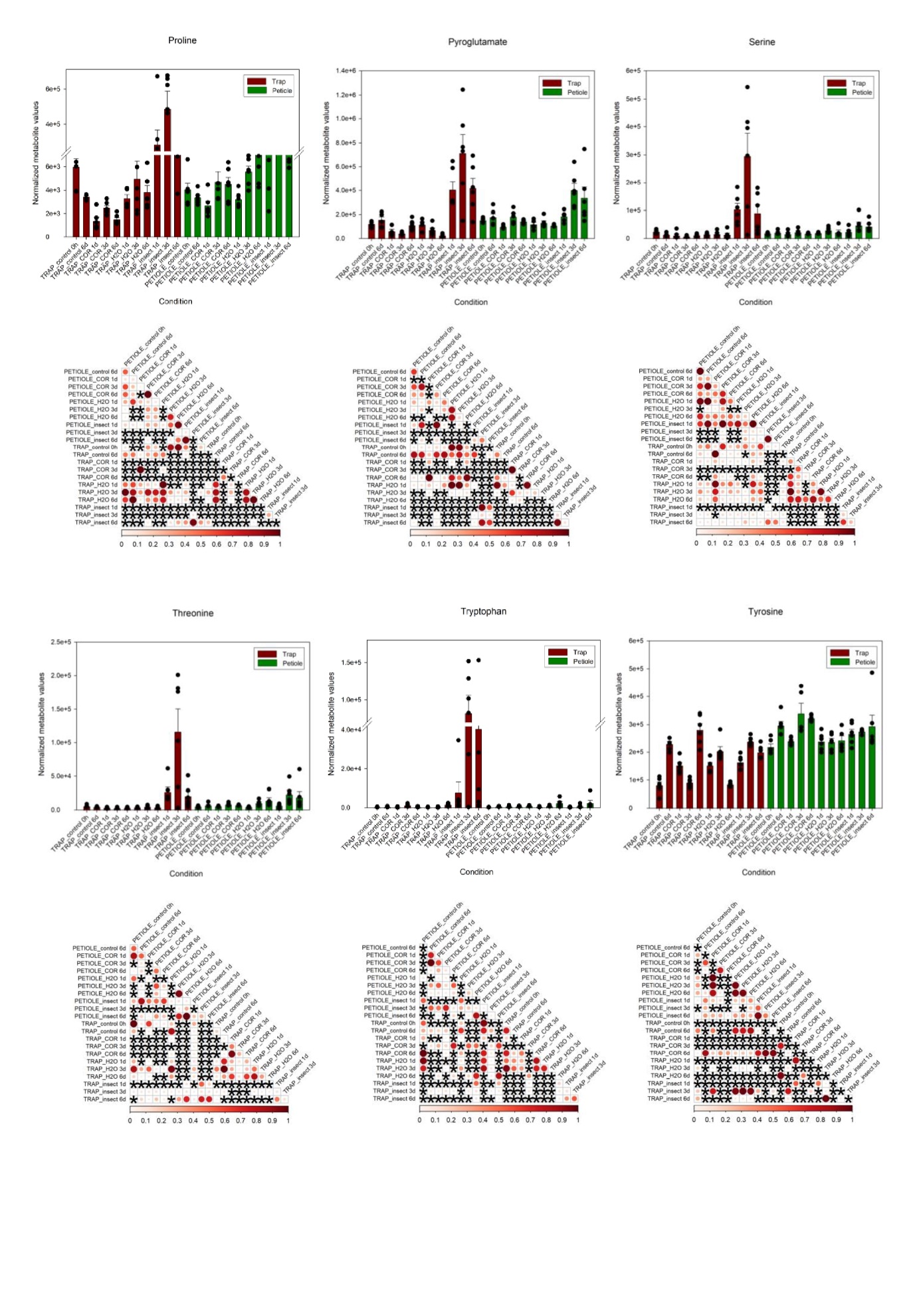

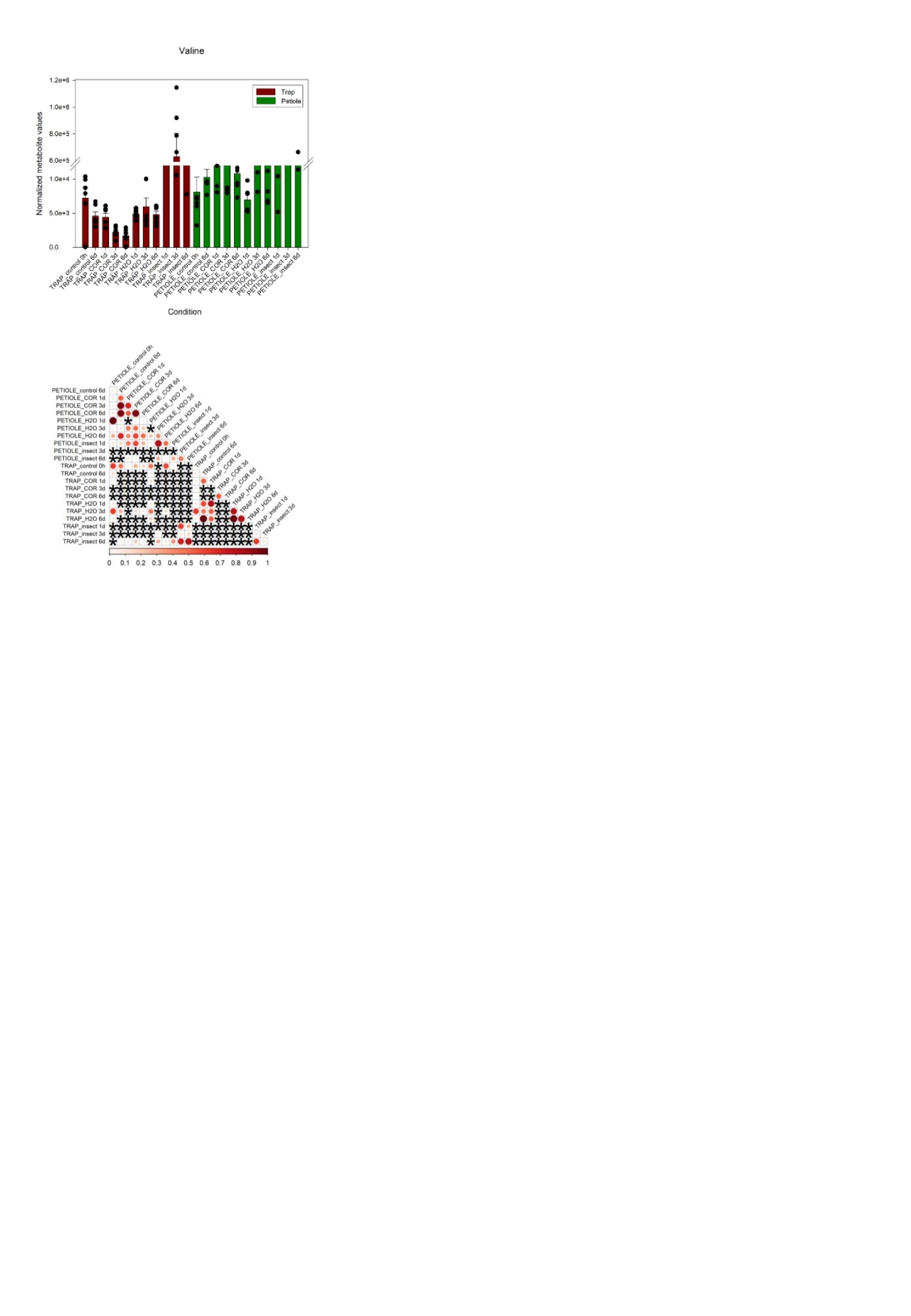

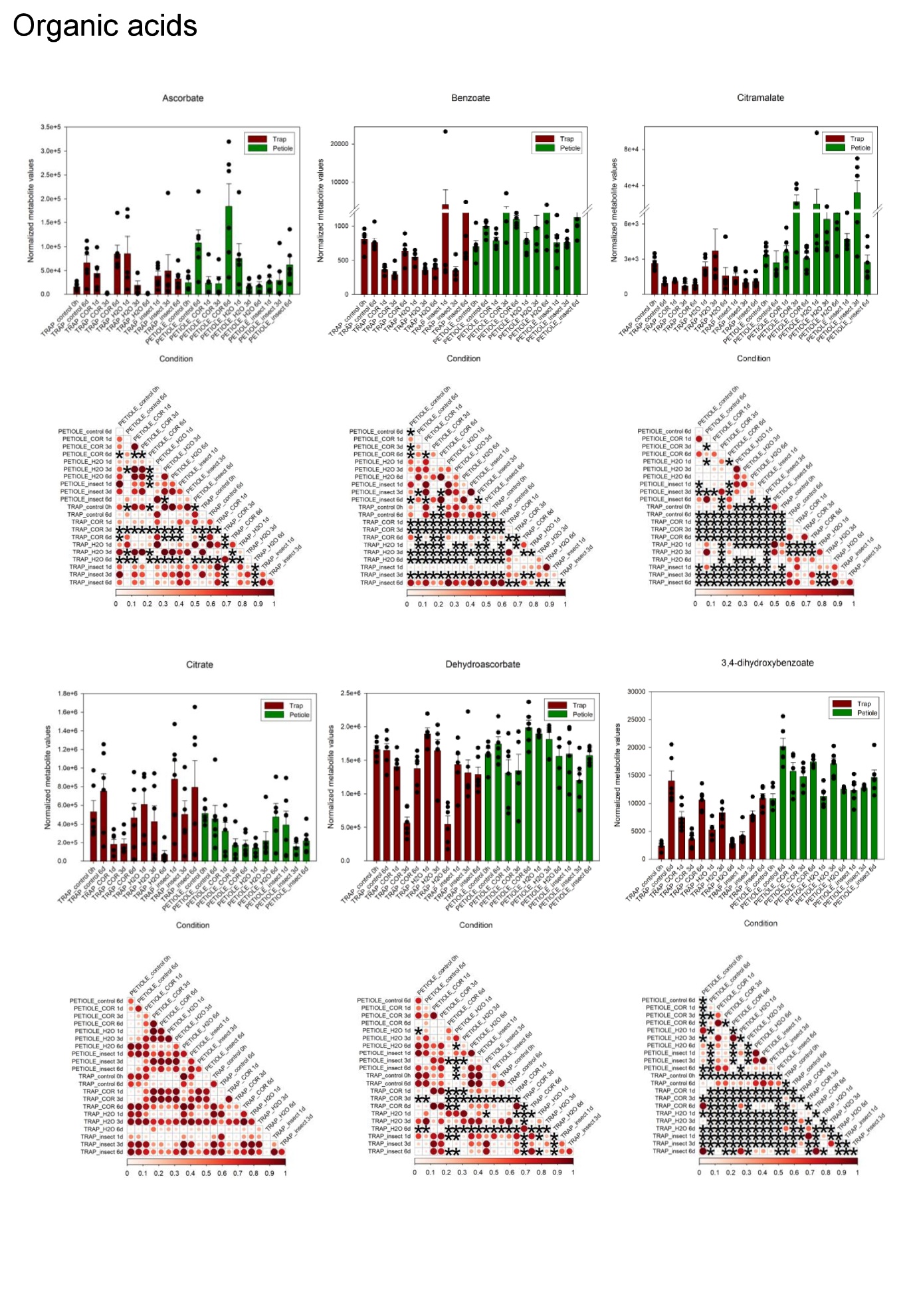

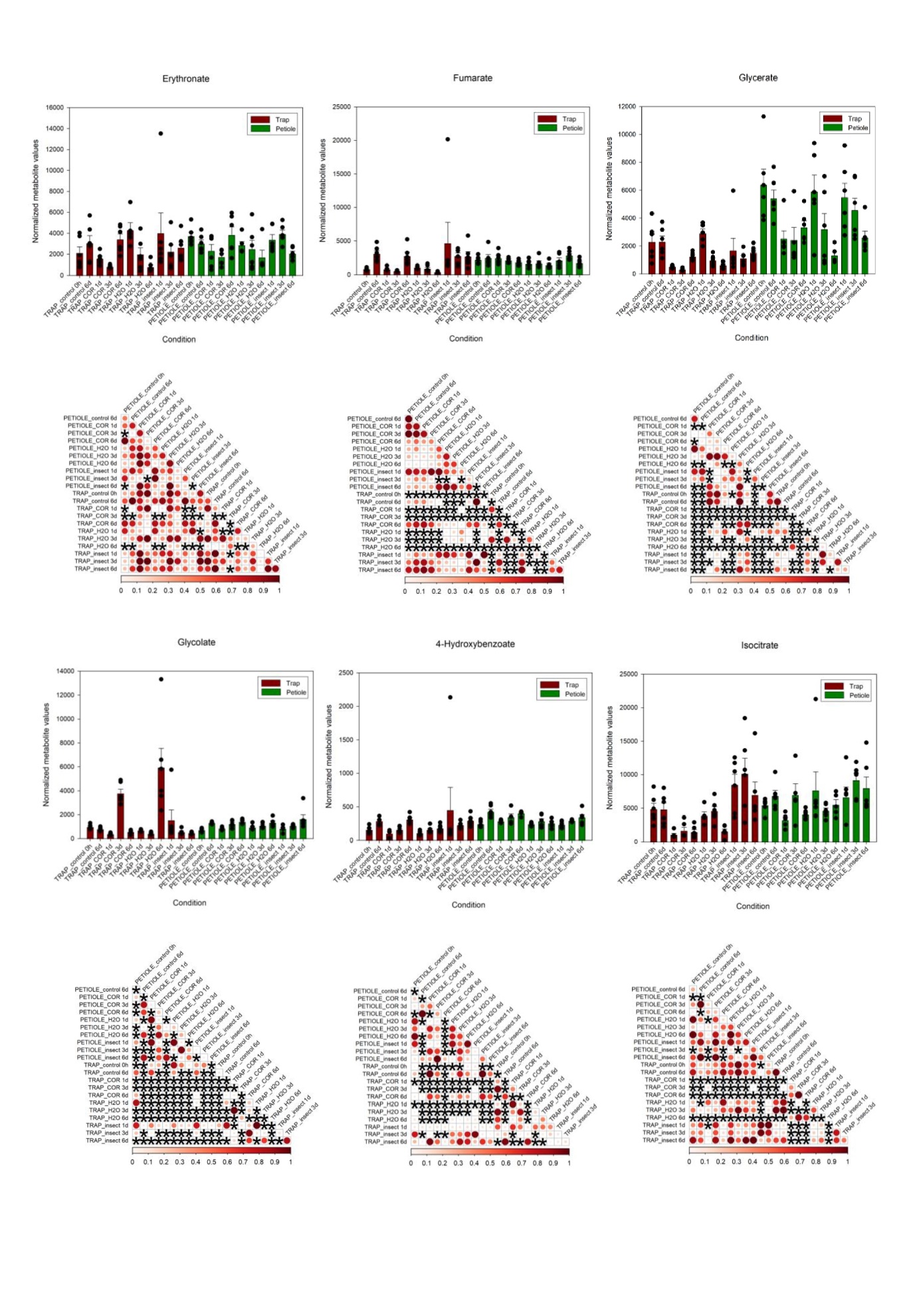

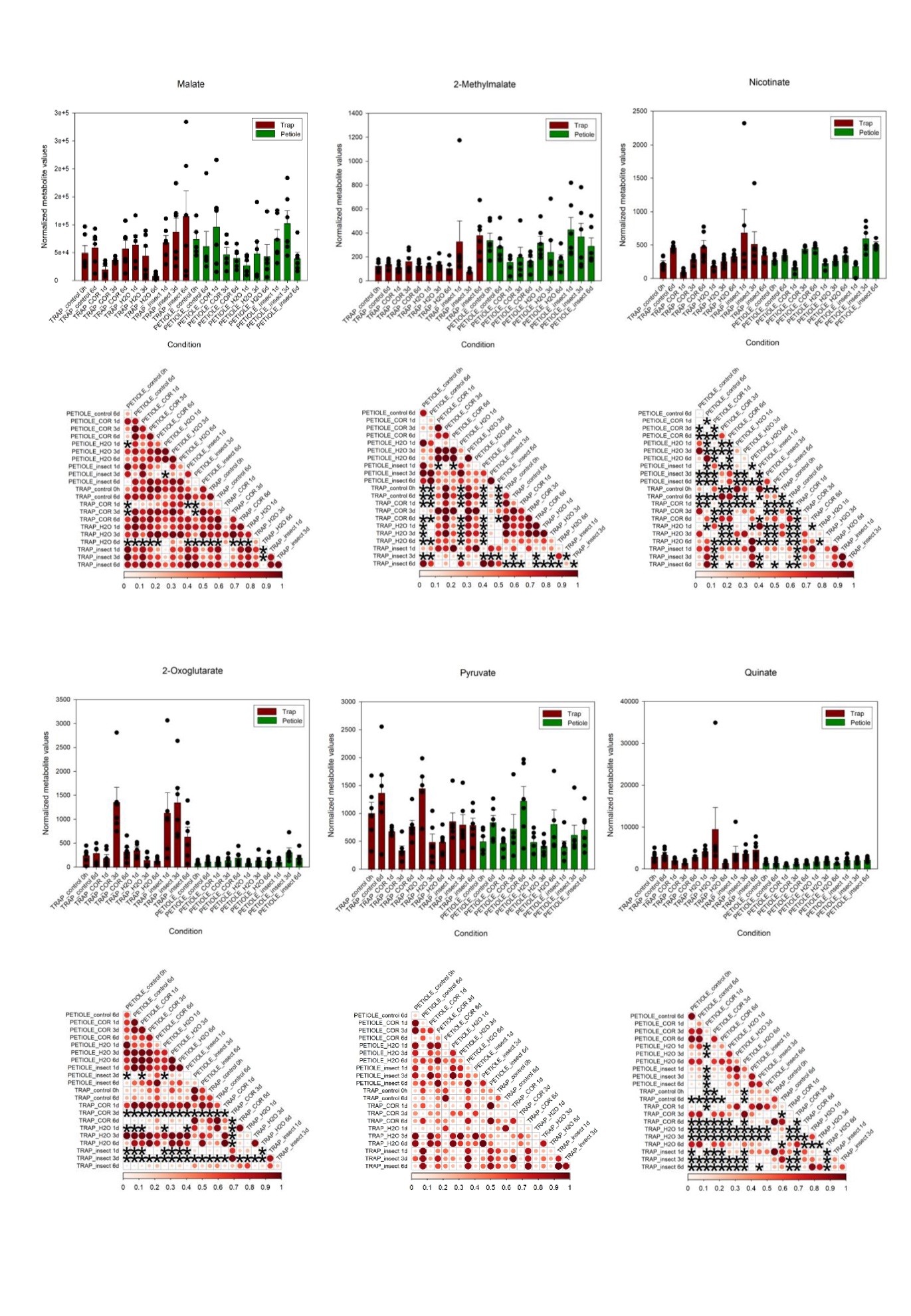

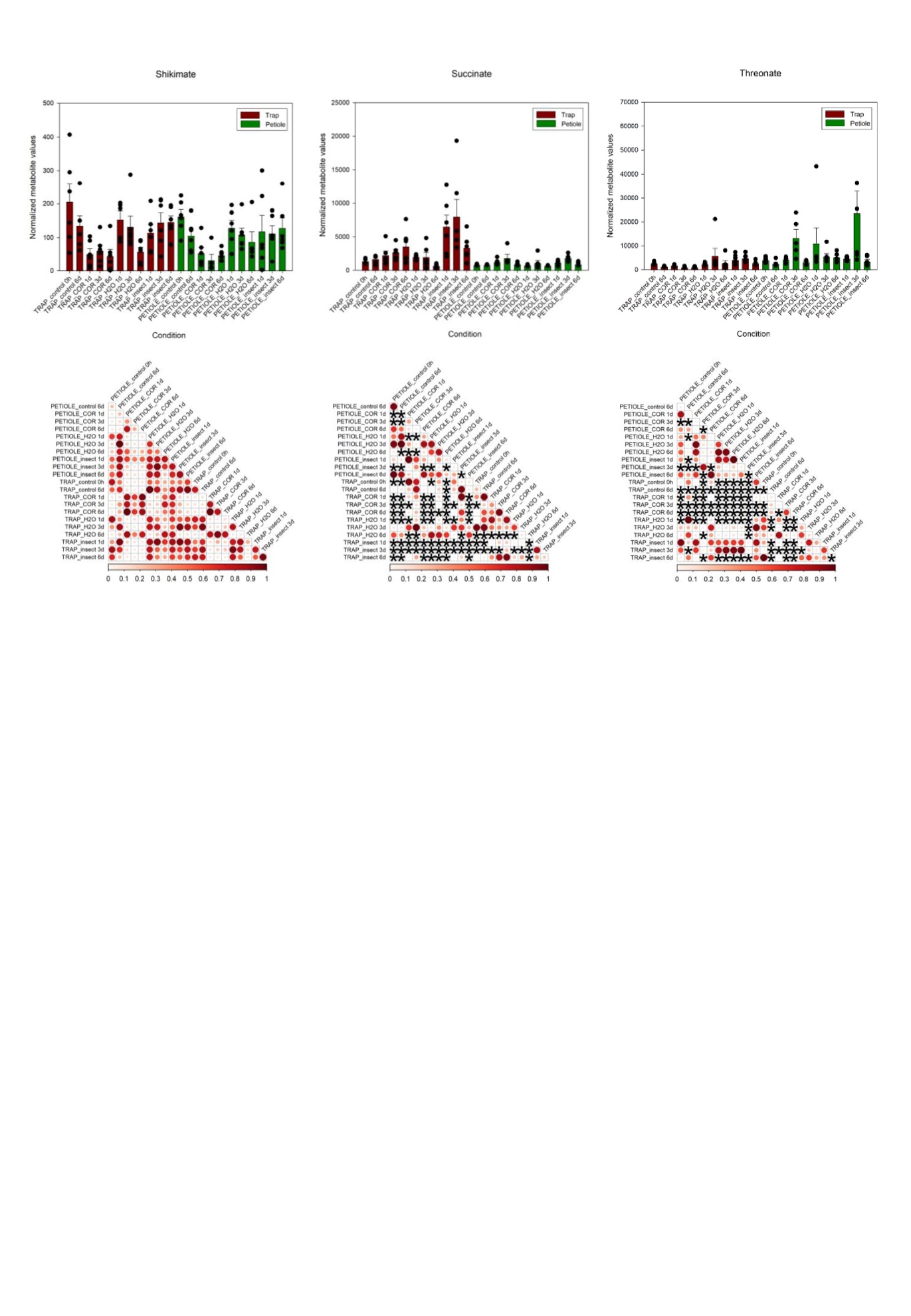

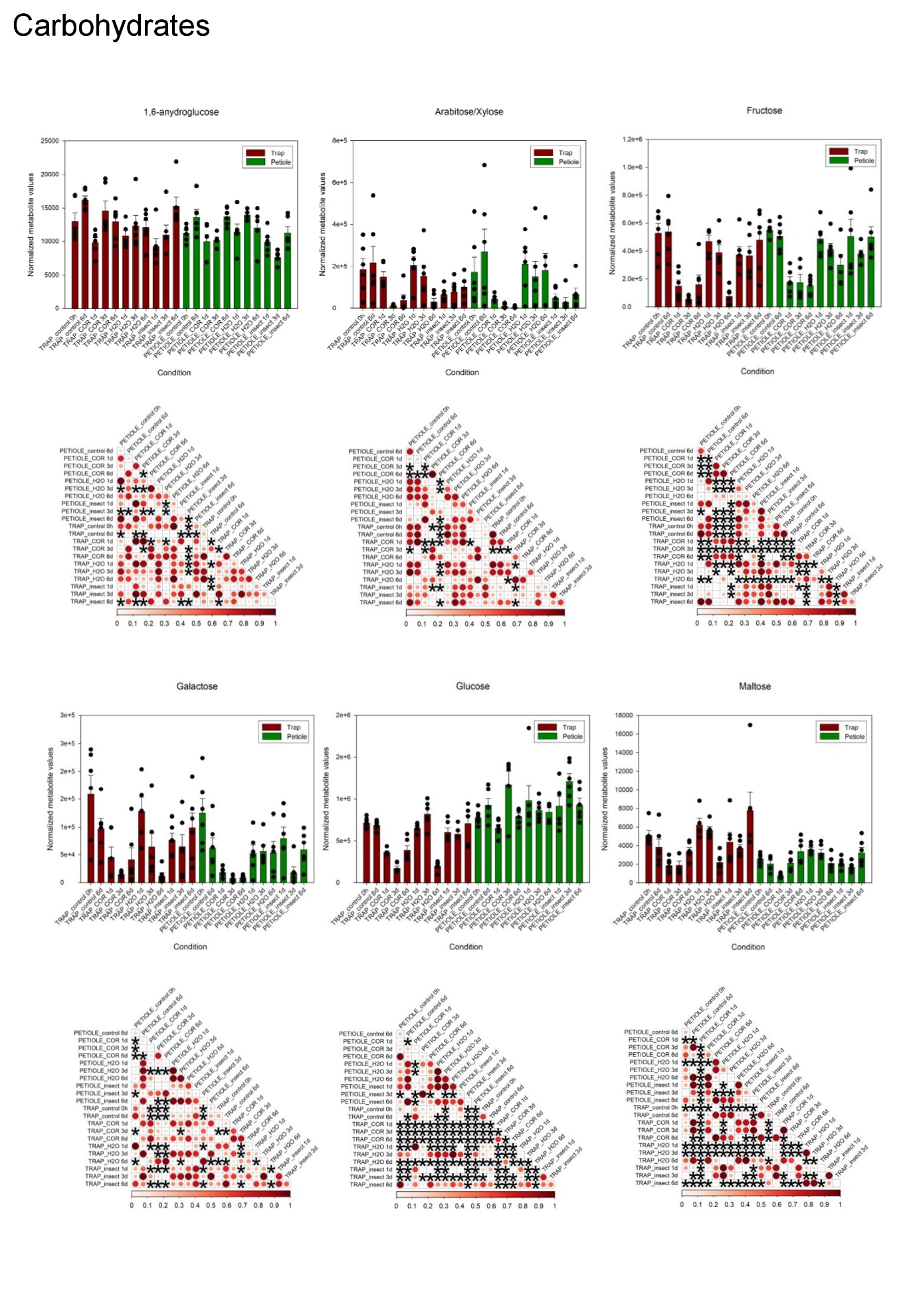

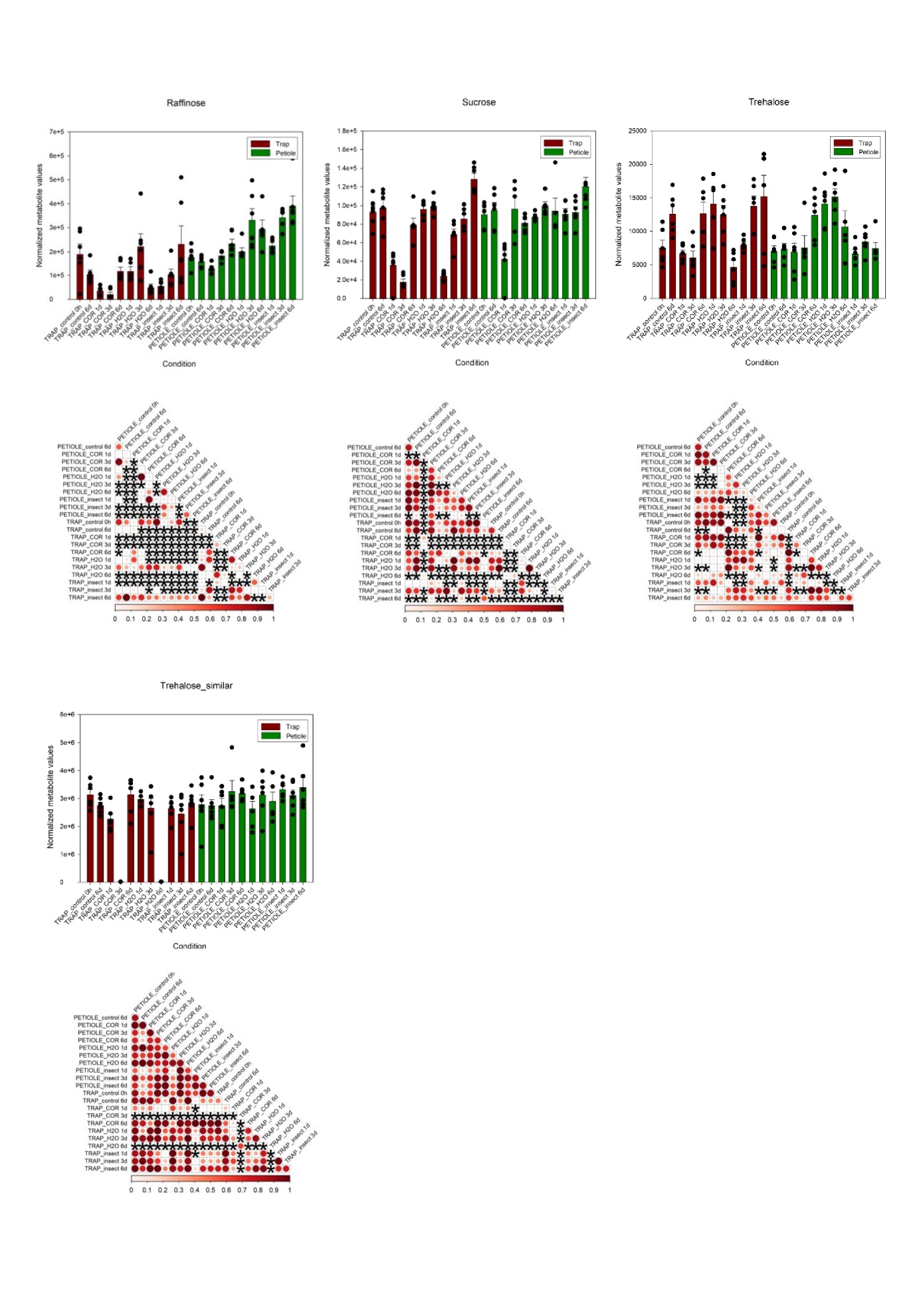

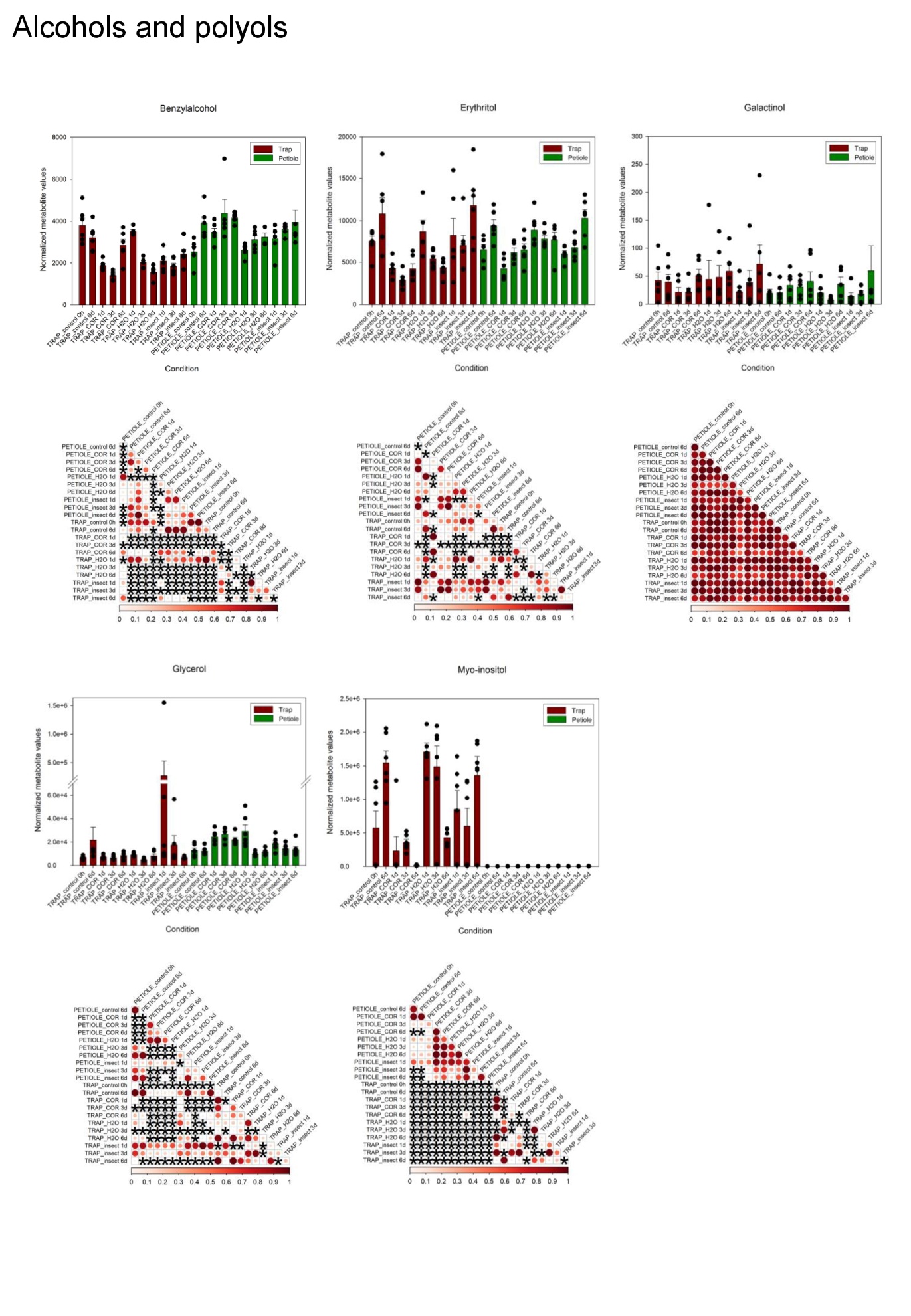

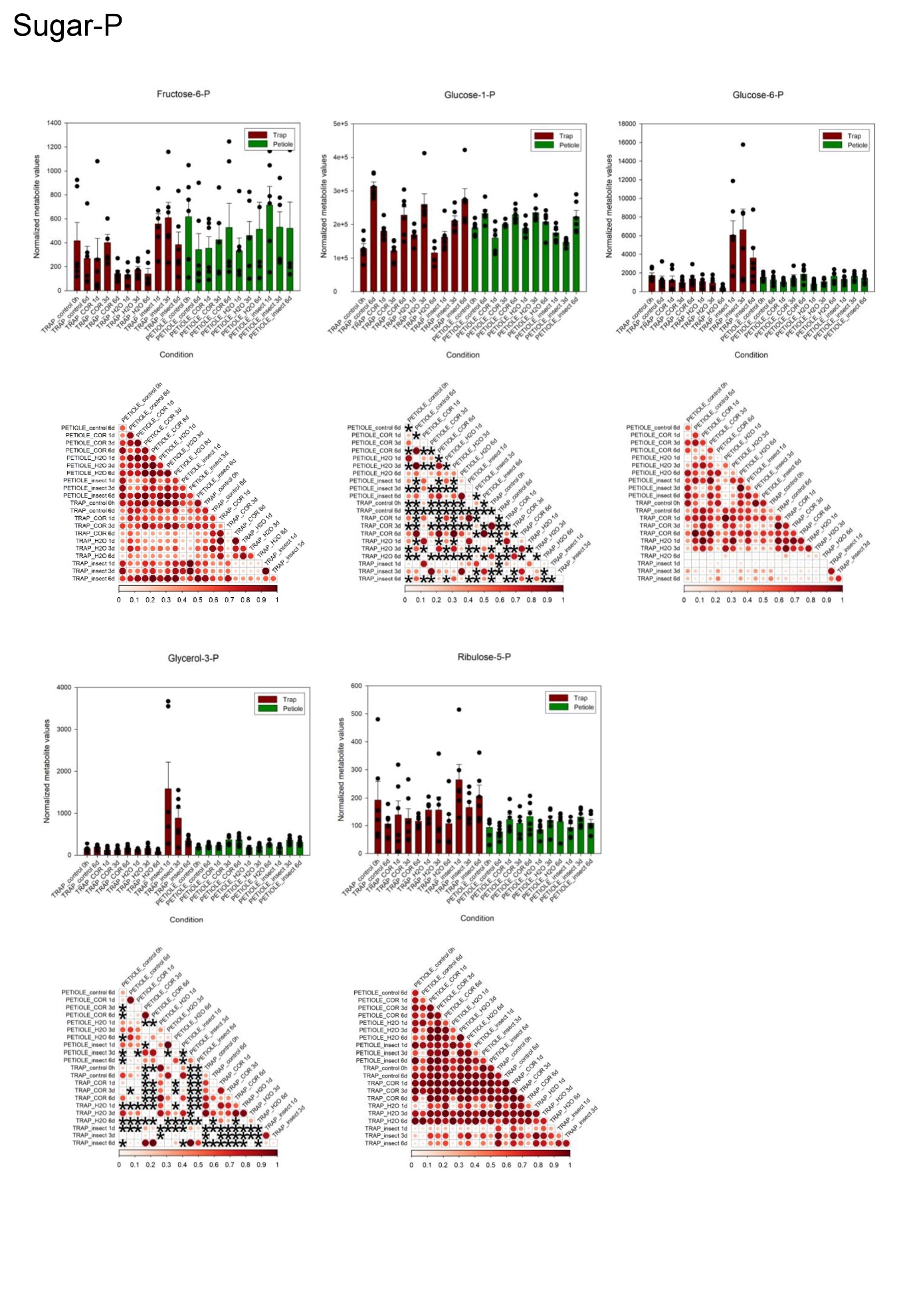

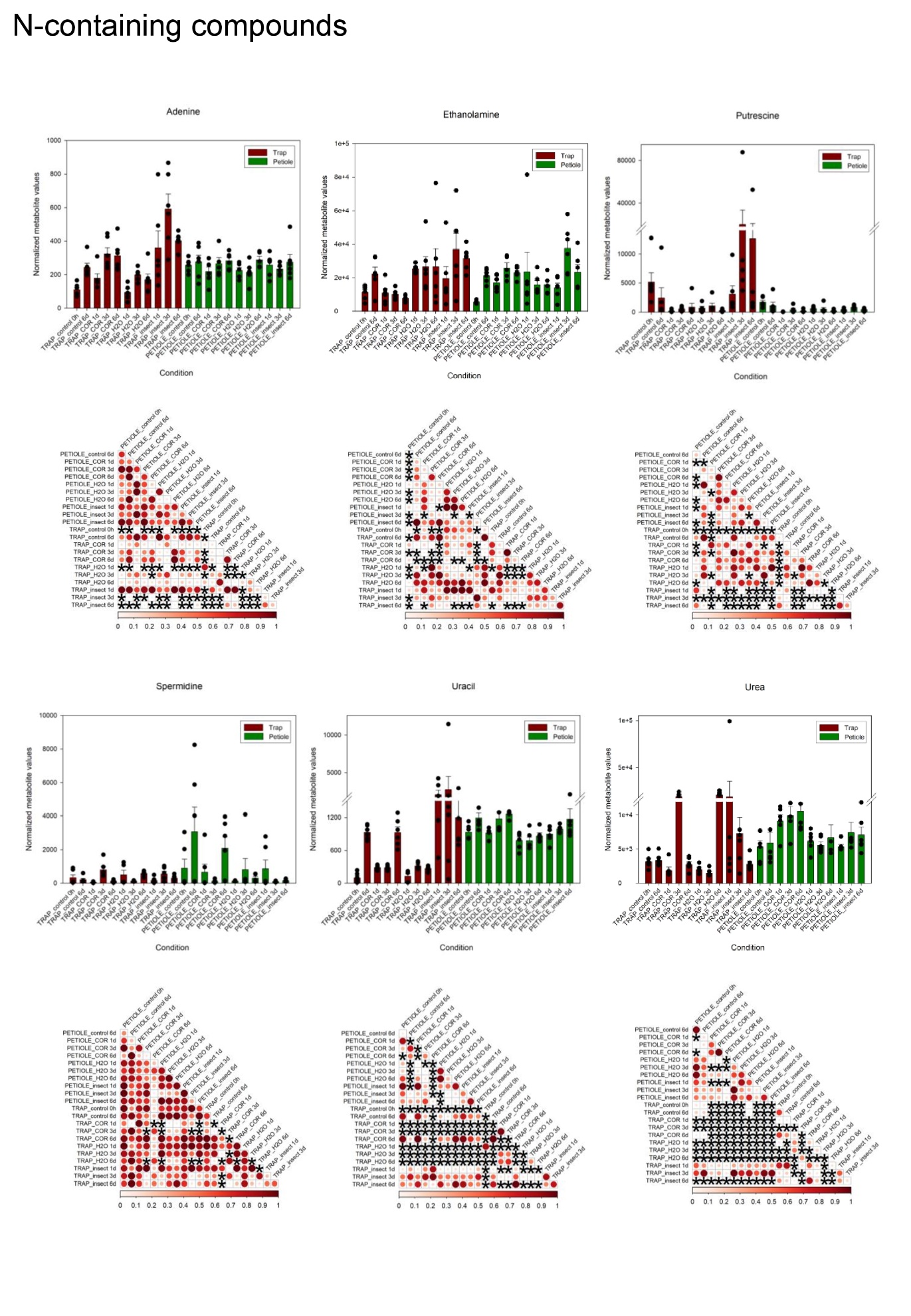

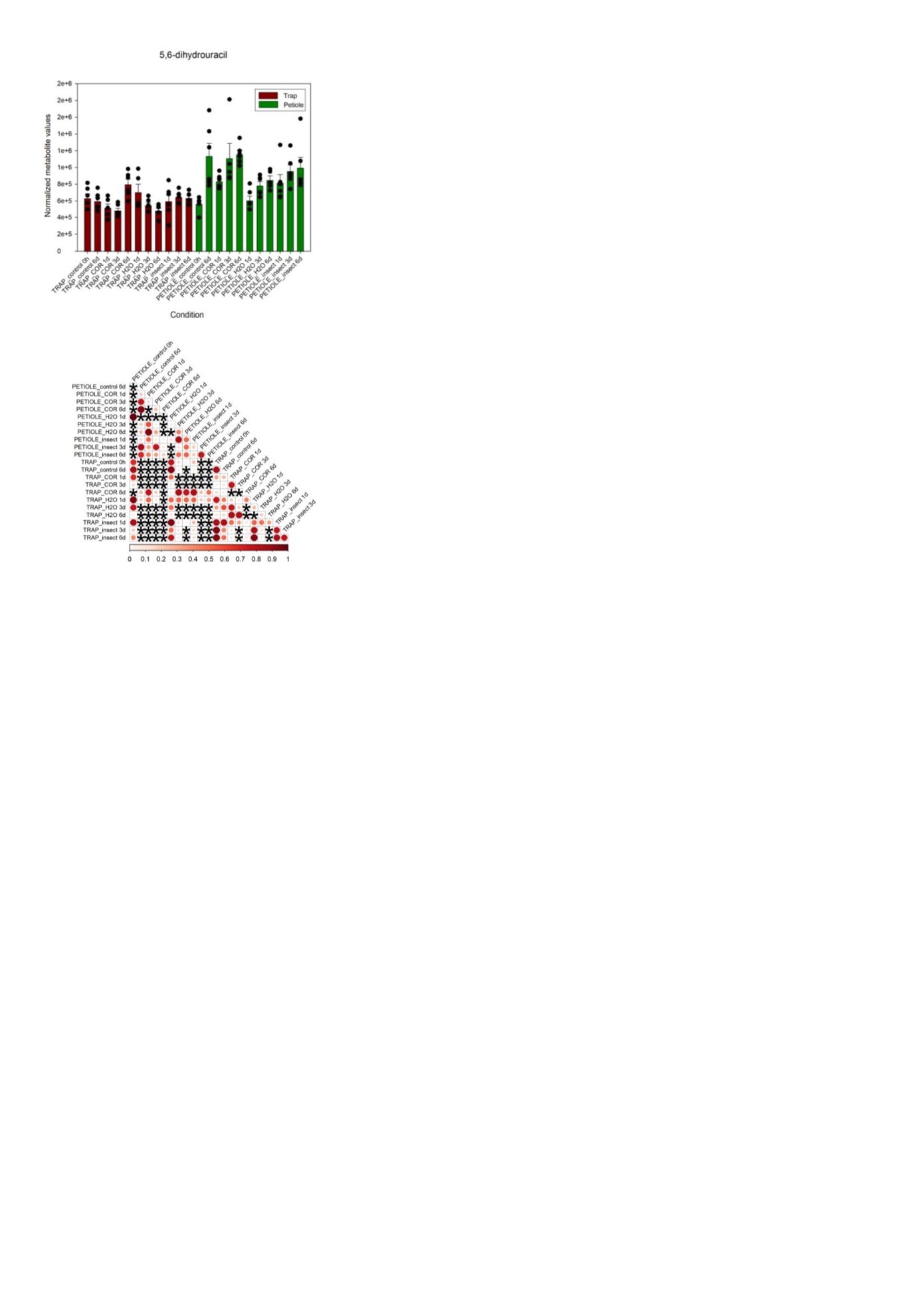

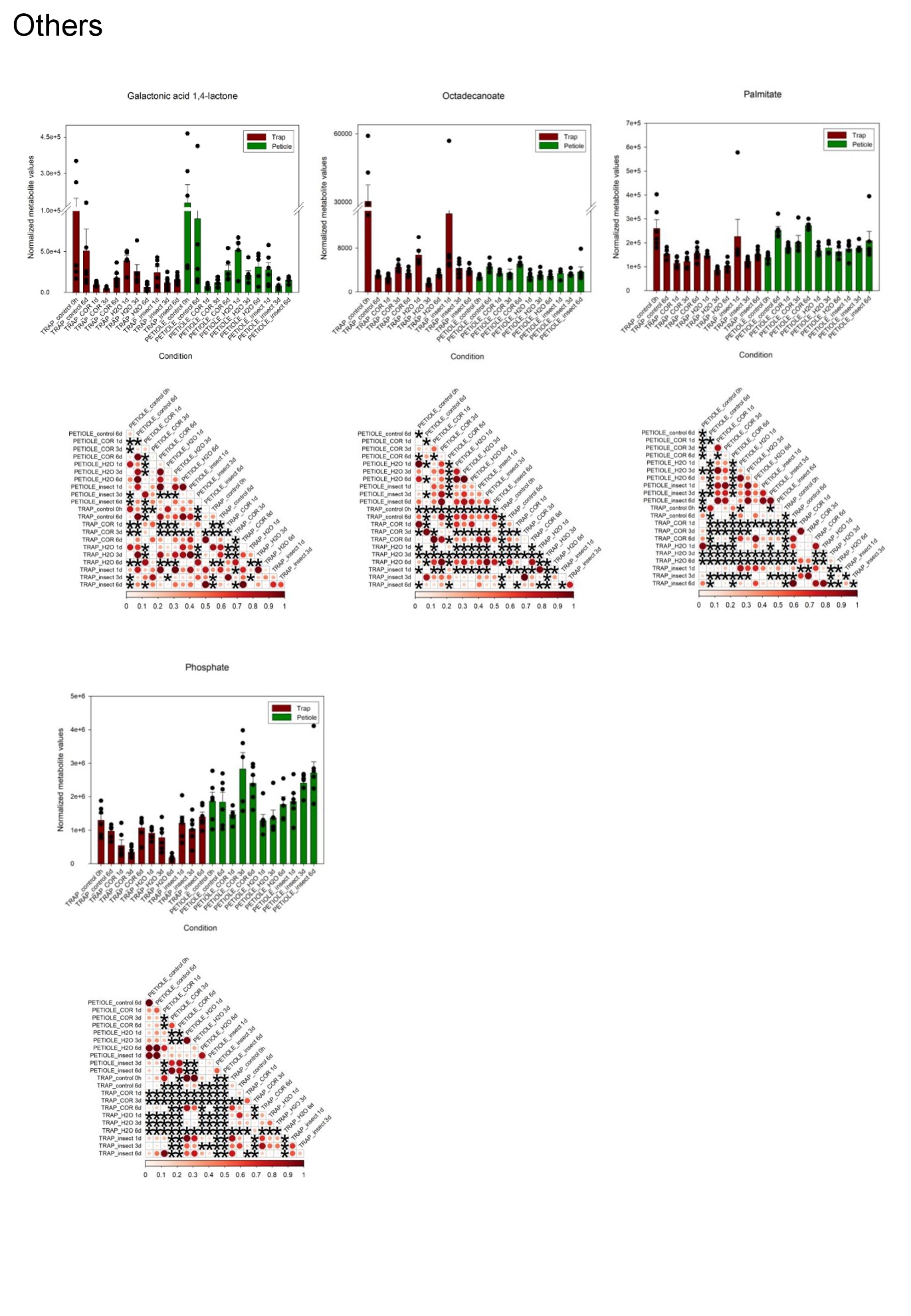


**Fig. S1. Histograms and associated matrices of p-values for the metabolites detected in the insect feeding experiment.** Data are expressed as average normalized metabolite values (a.u.) ± SE. The triangular matrices below each histogram represent the significance of the p-values for all pairwise combinations. An asterisk denotes a significant difference between two conditions (adj pval < 0.05), while red circles denote a non-significant difference (with darker reds for comparisons approaching a p-value of 1). The conditions denoted as “TRAP/PETIOLE_H2O_1/3/6d” refer to mechanostimulated (water-treated) traps and associated petioles.


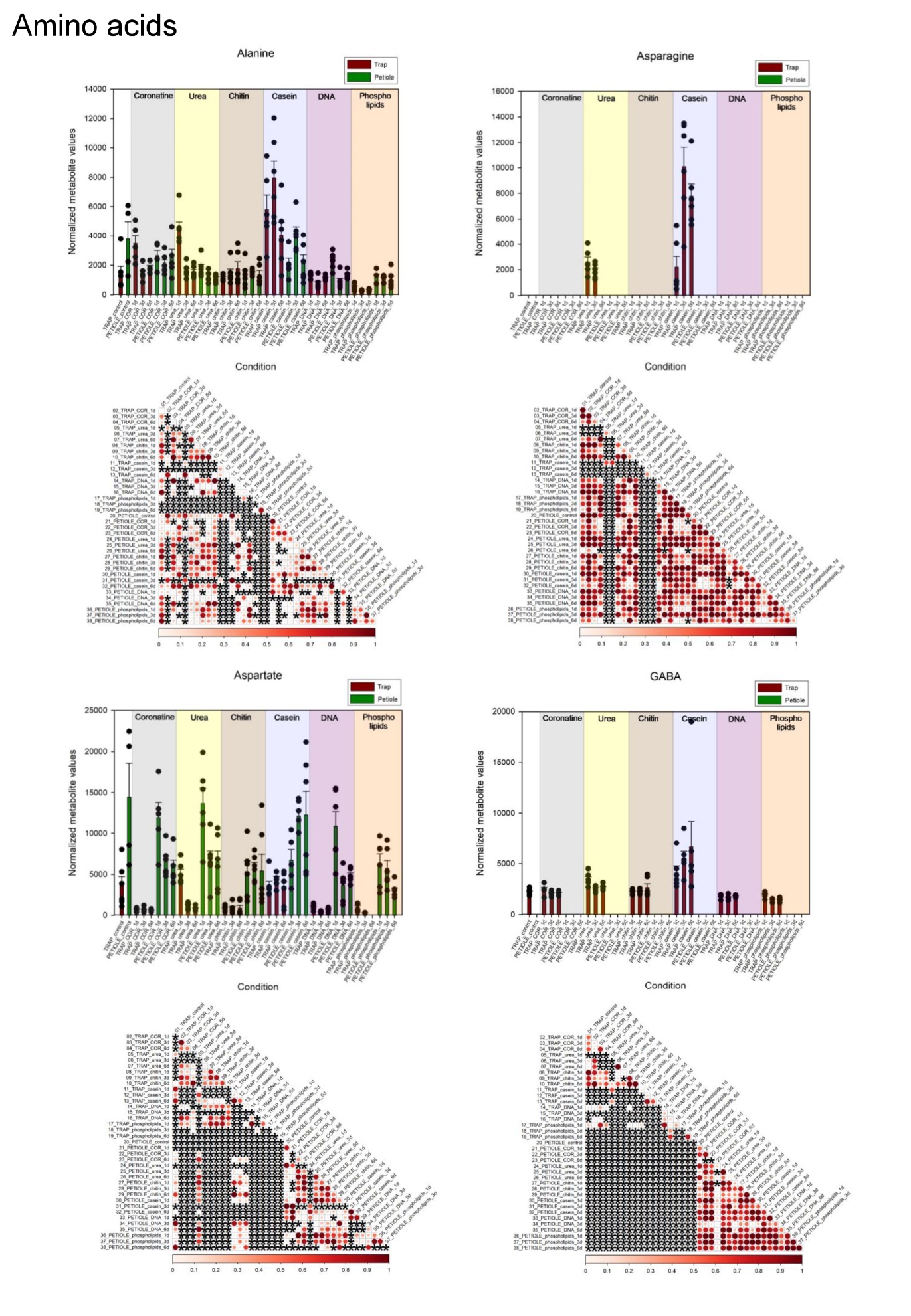

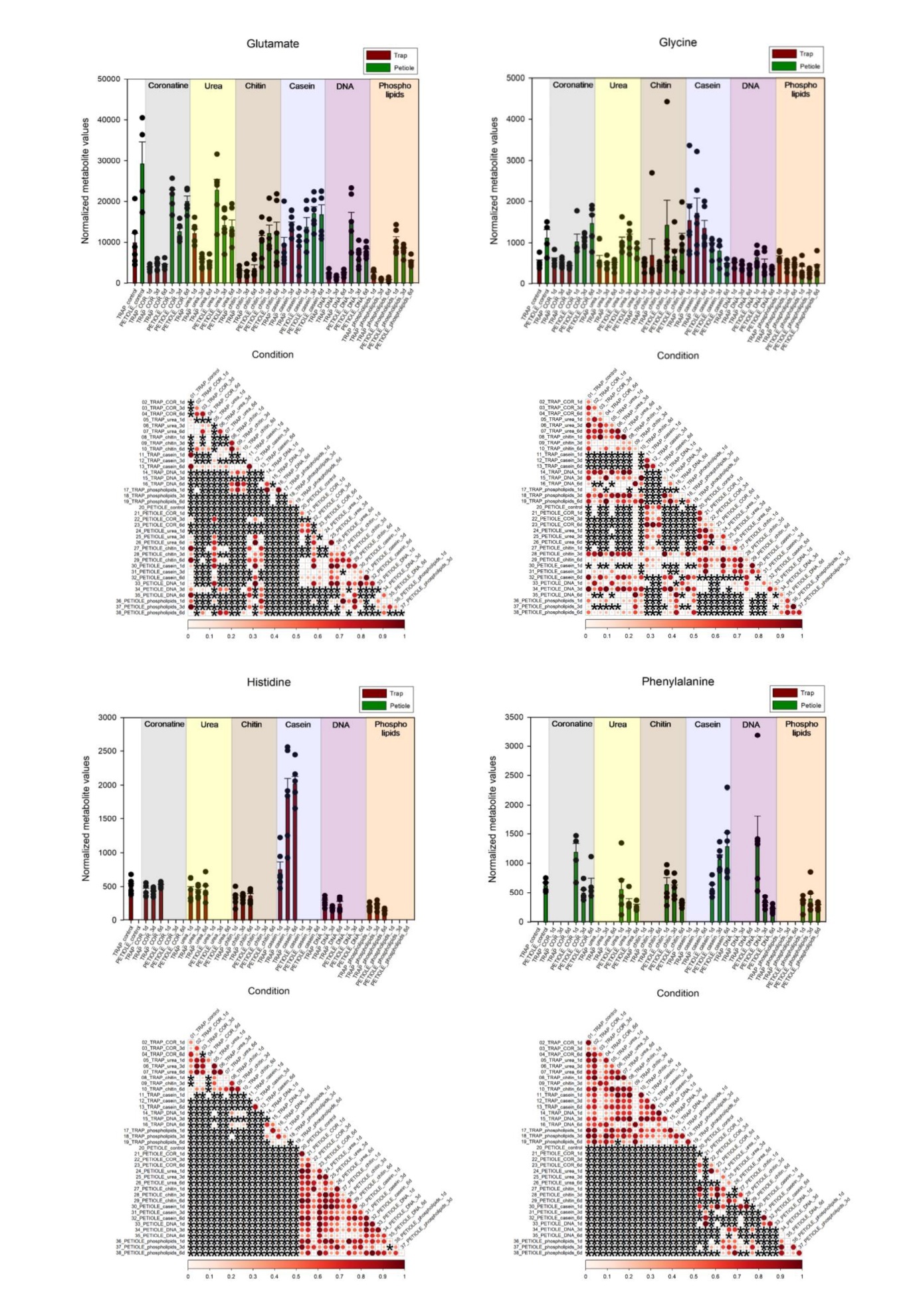

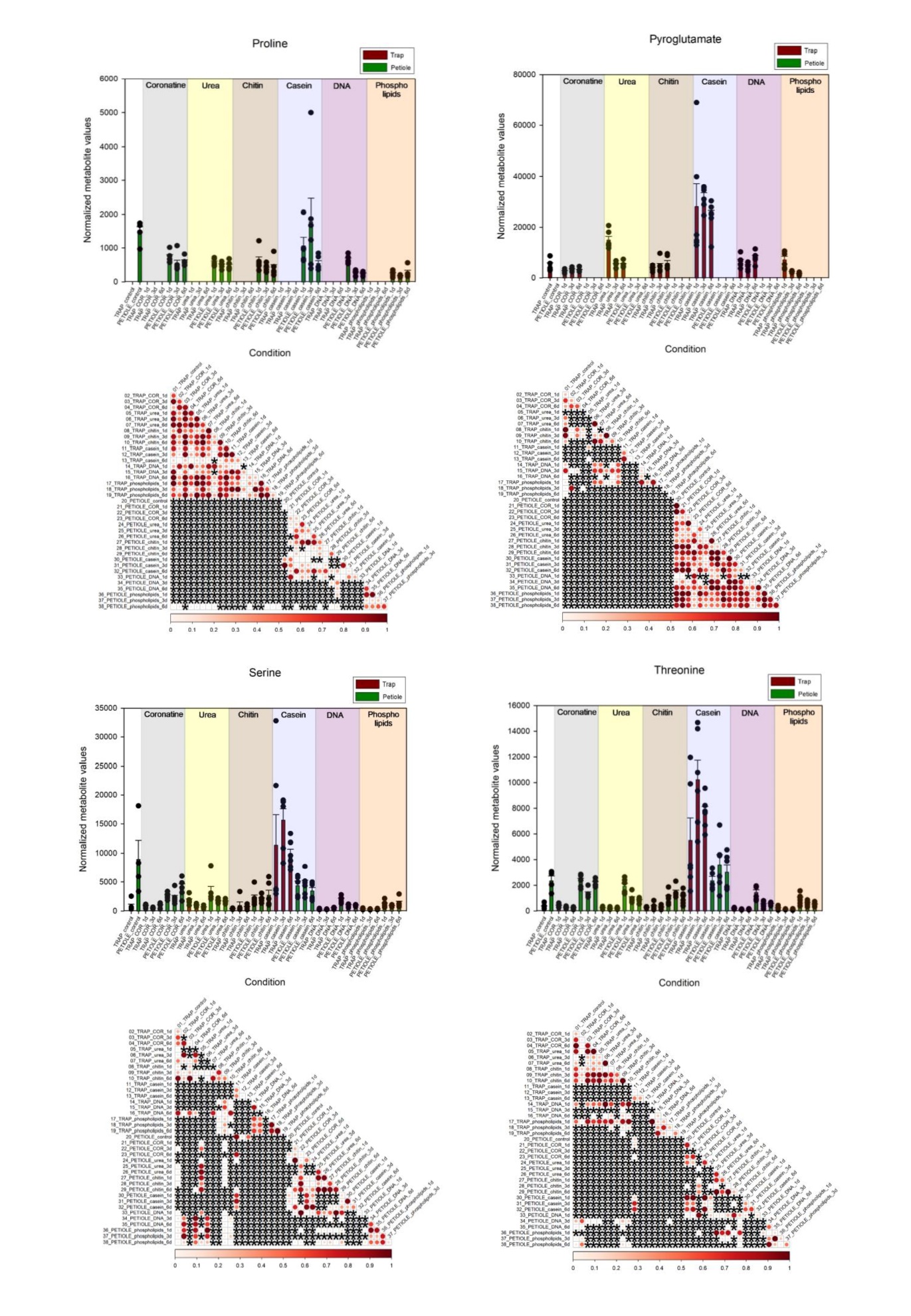

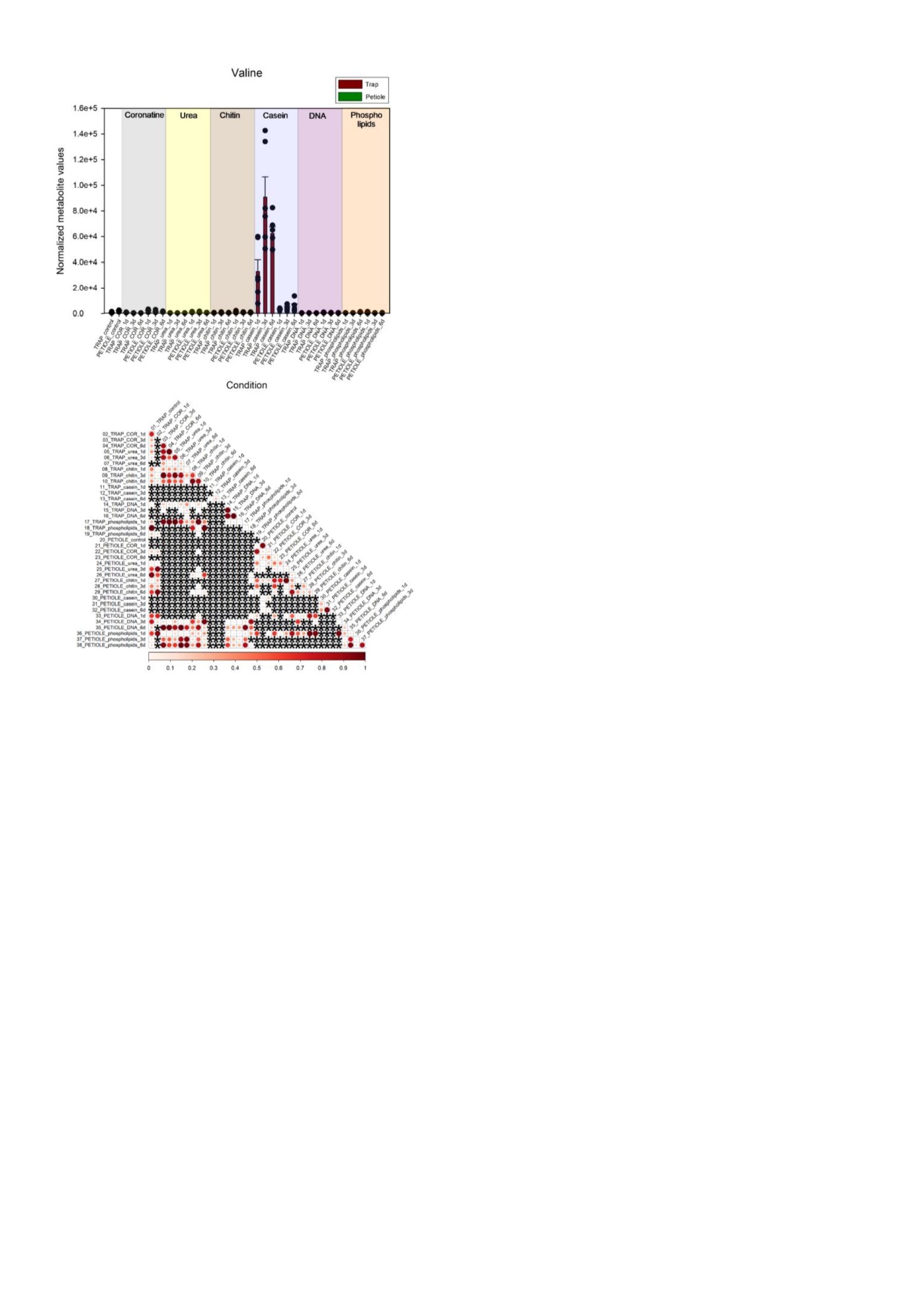

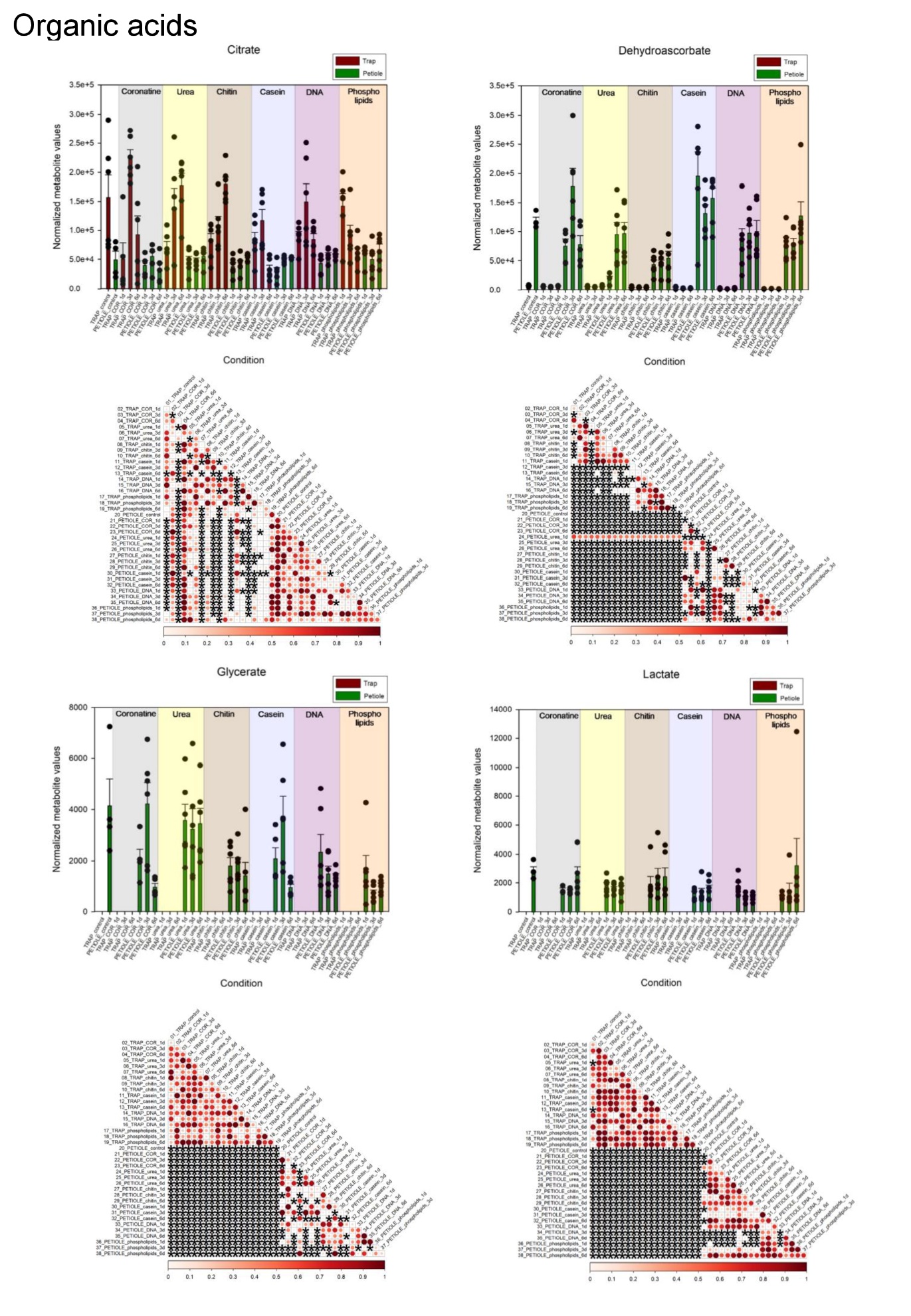

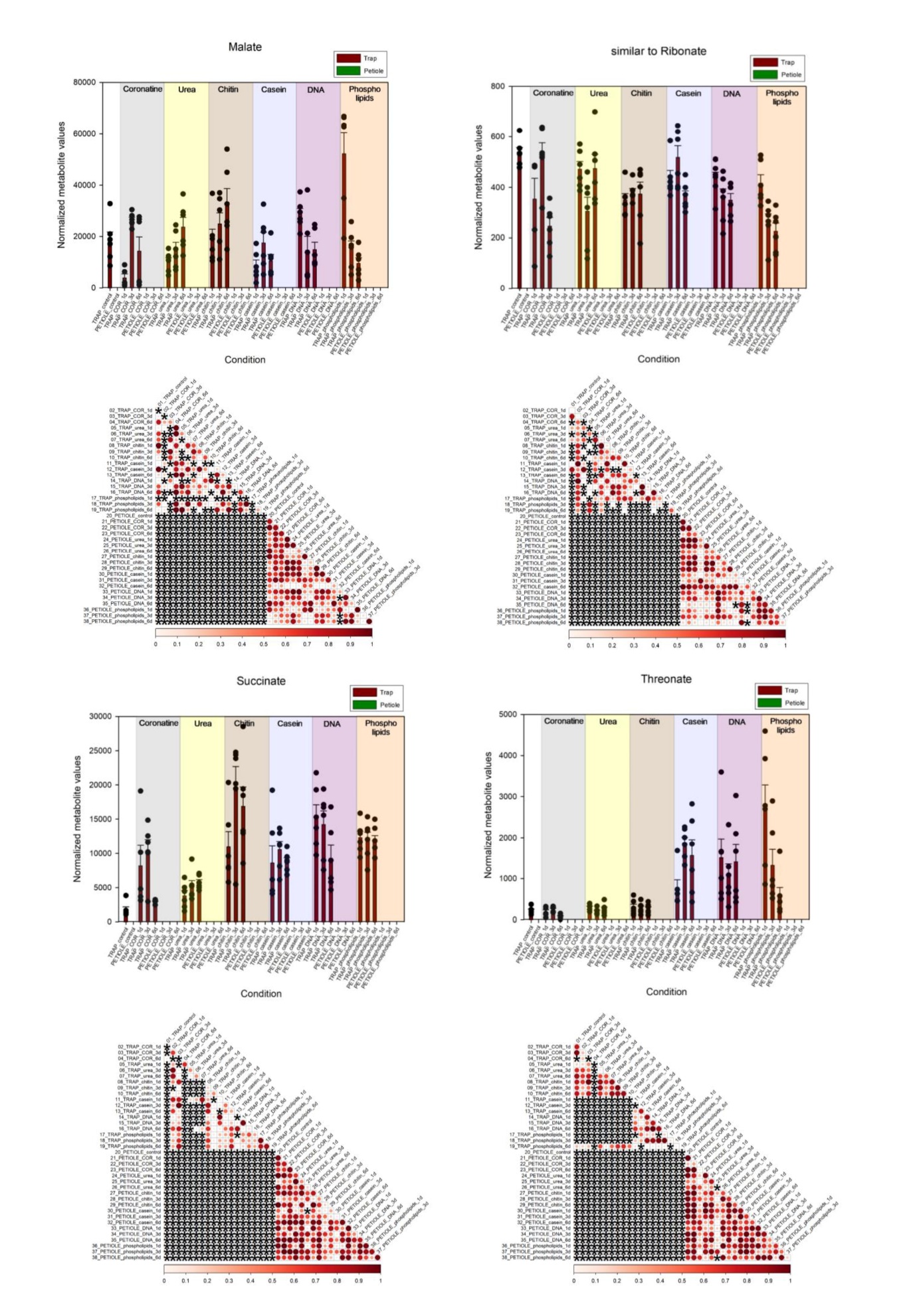

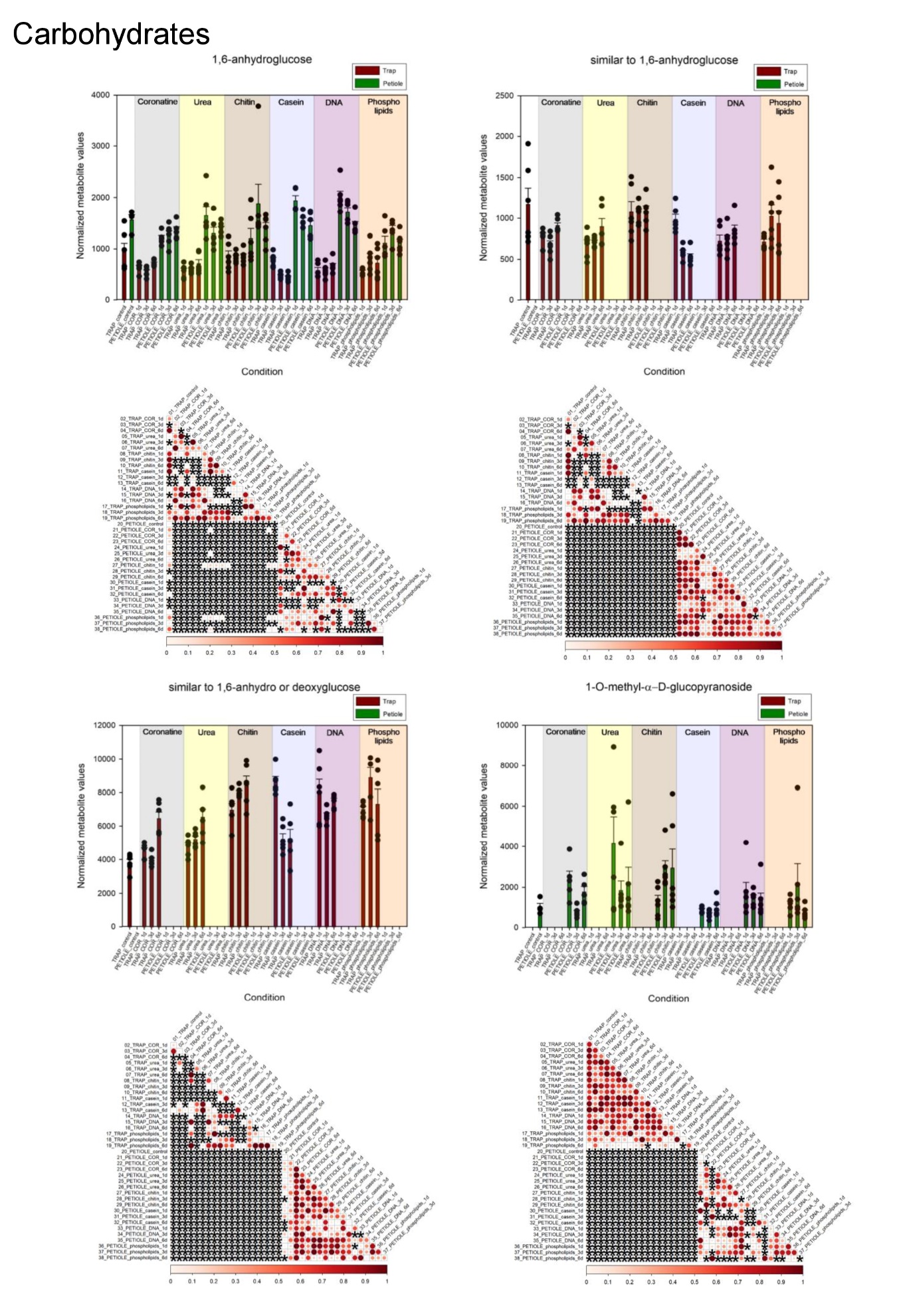

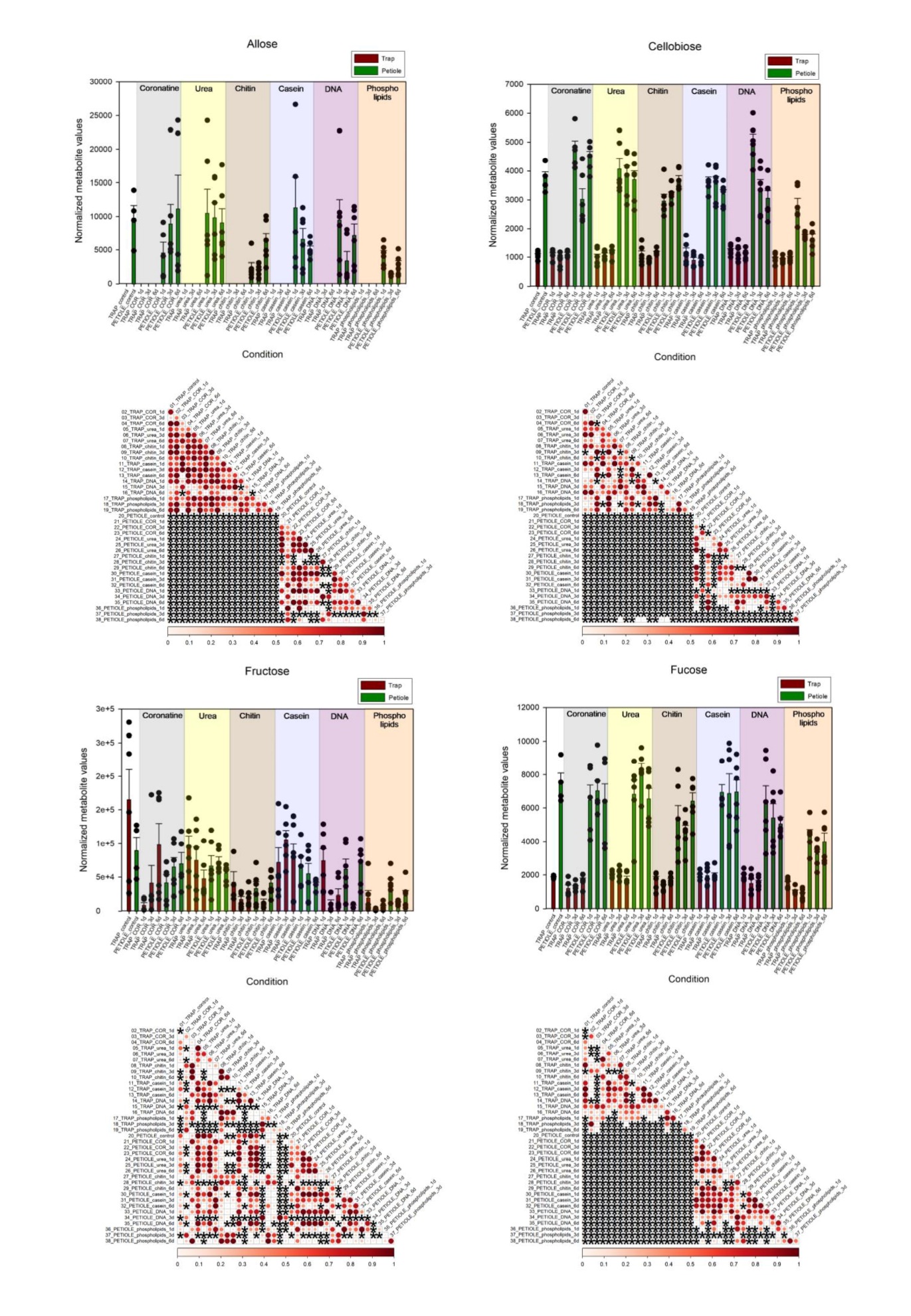

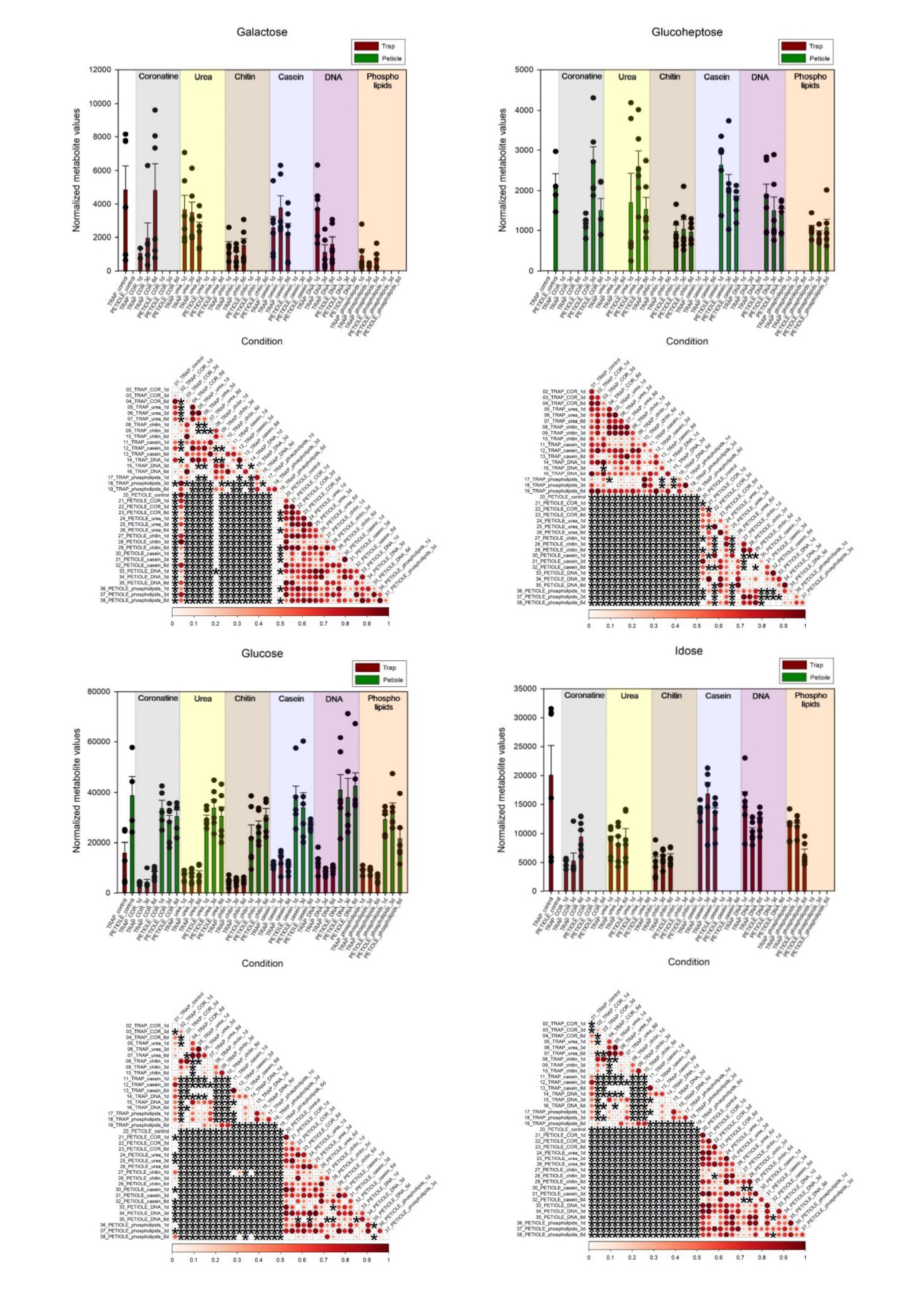

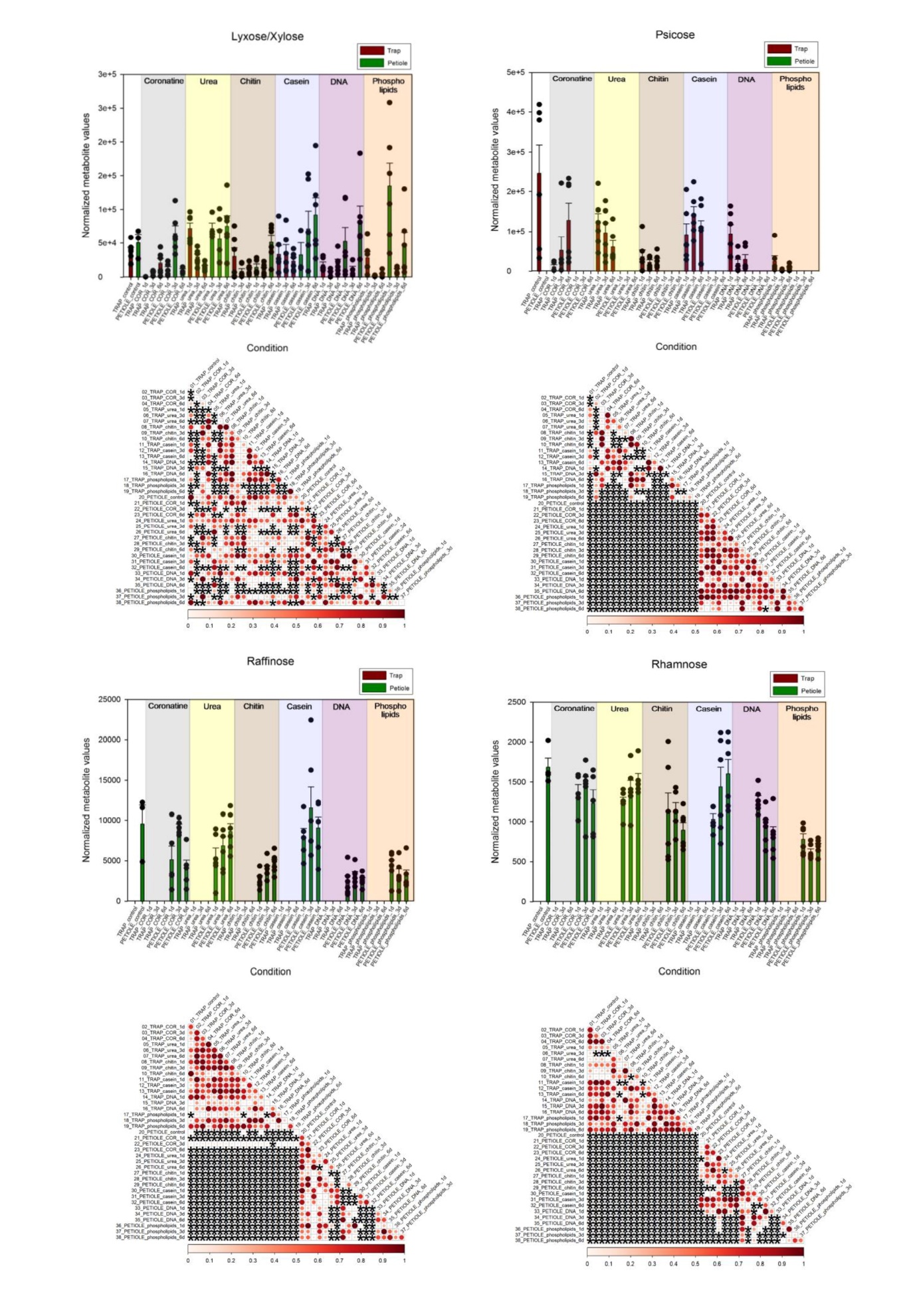

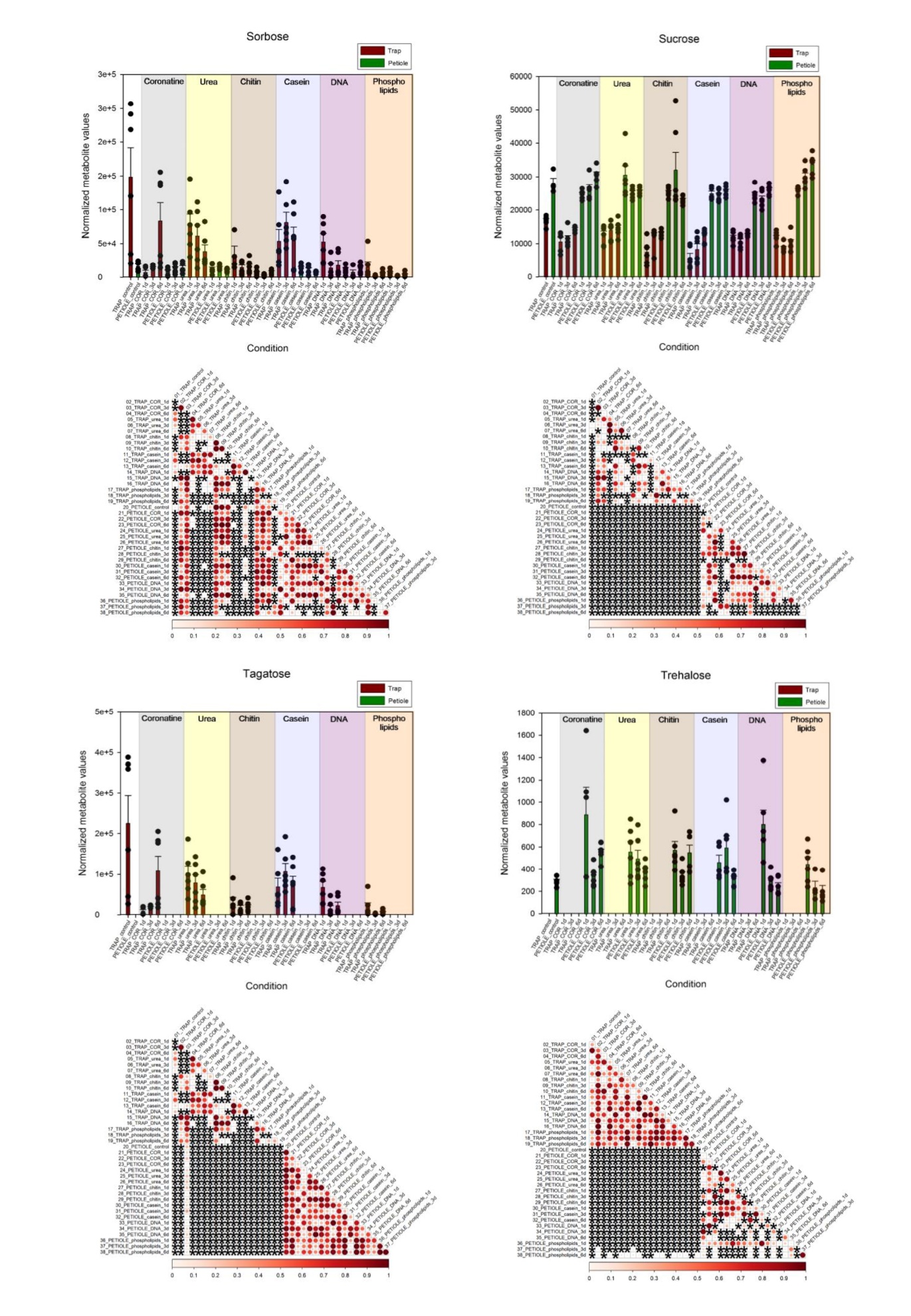

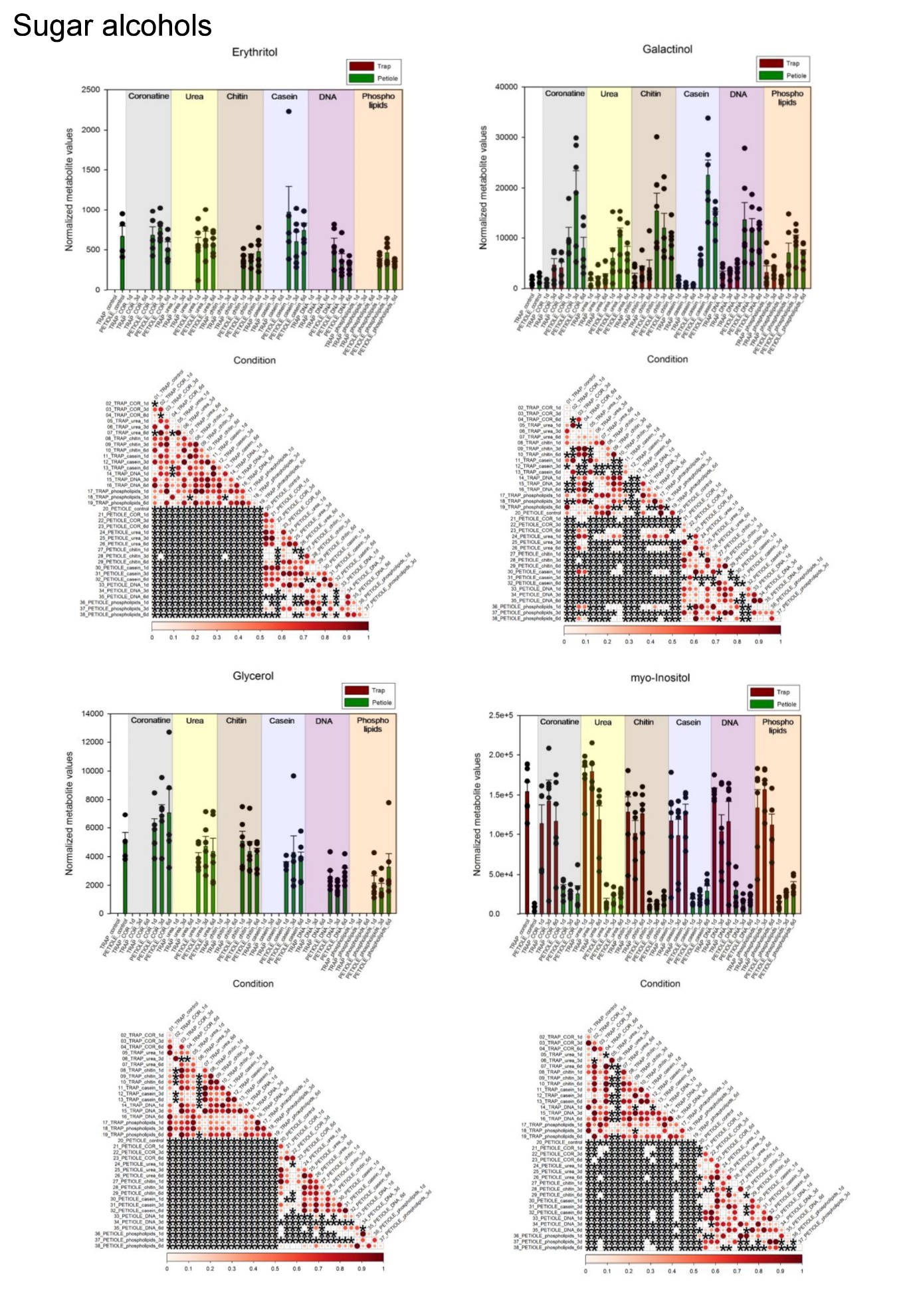

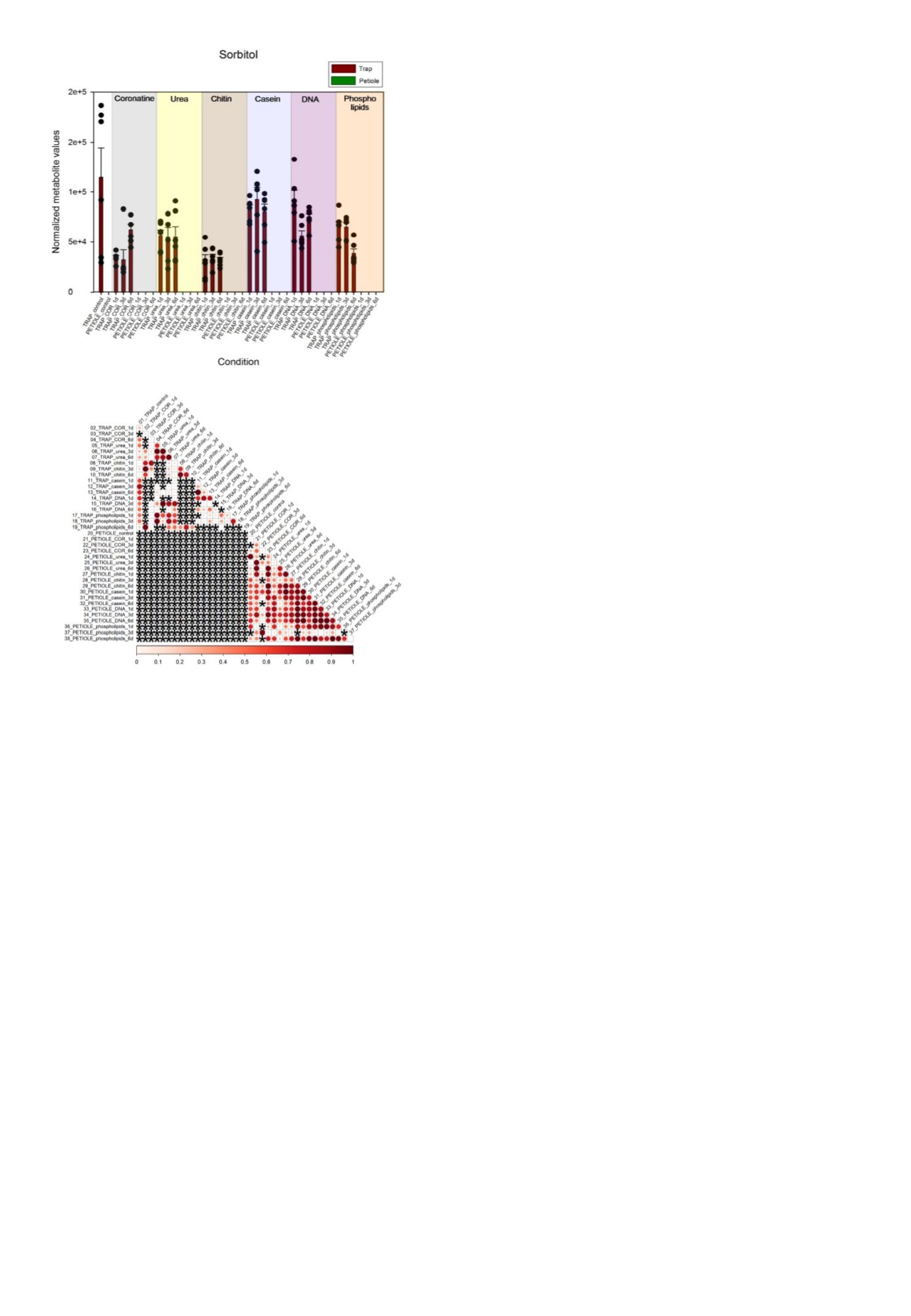

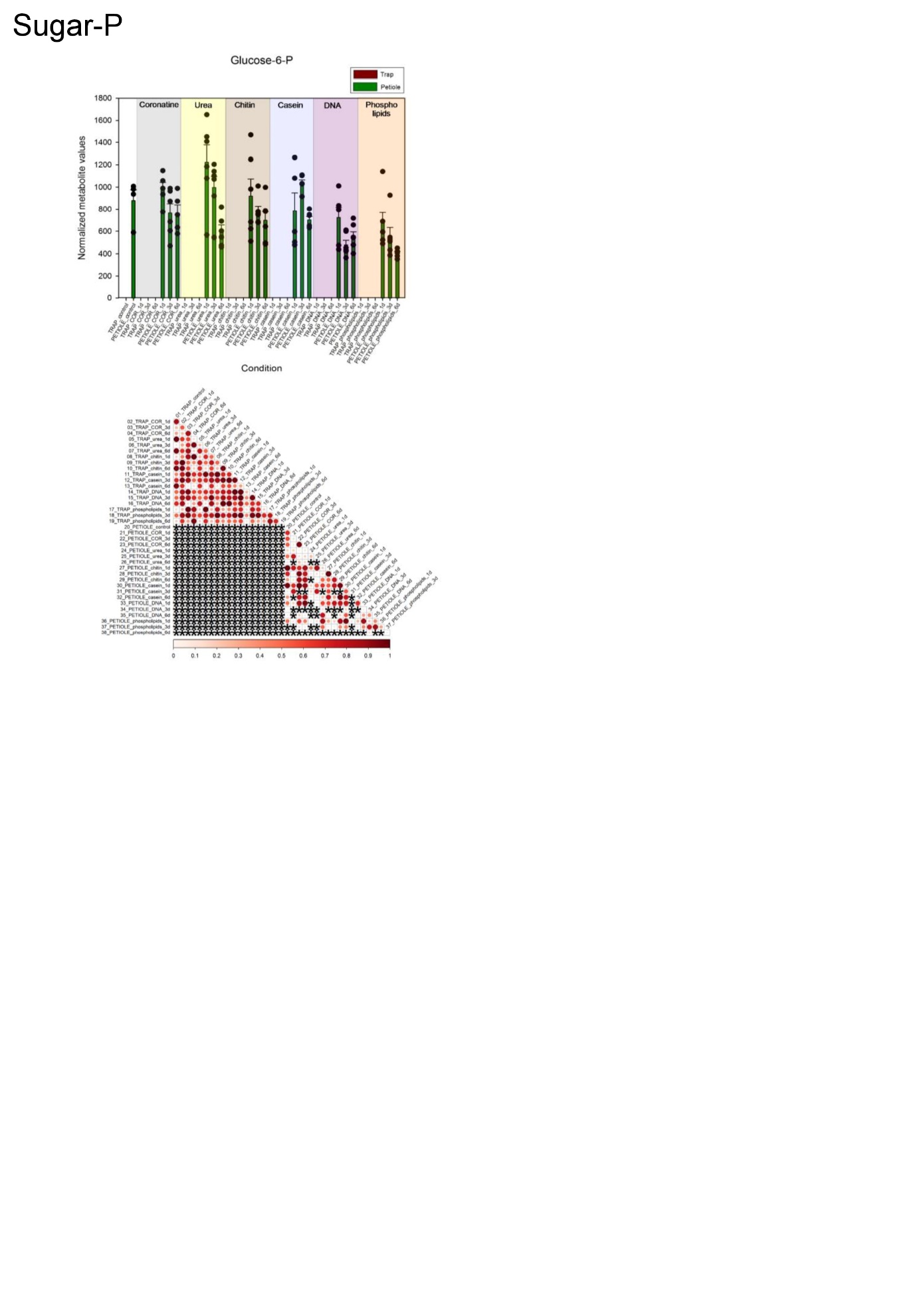

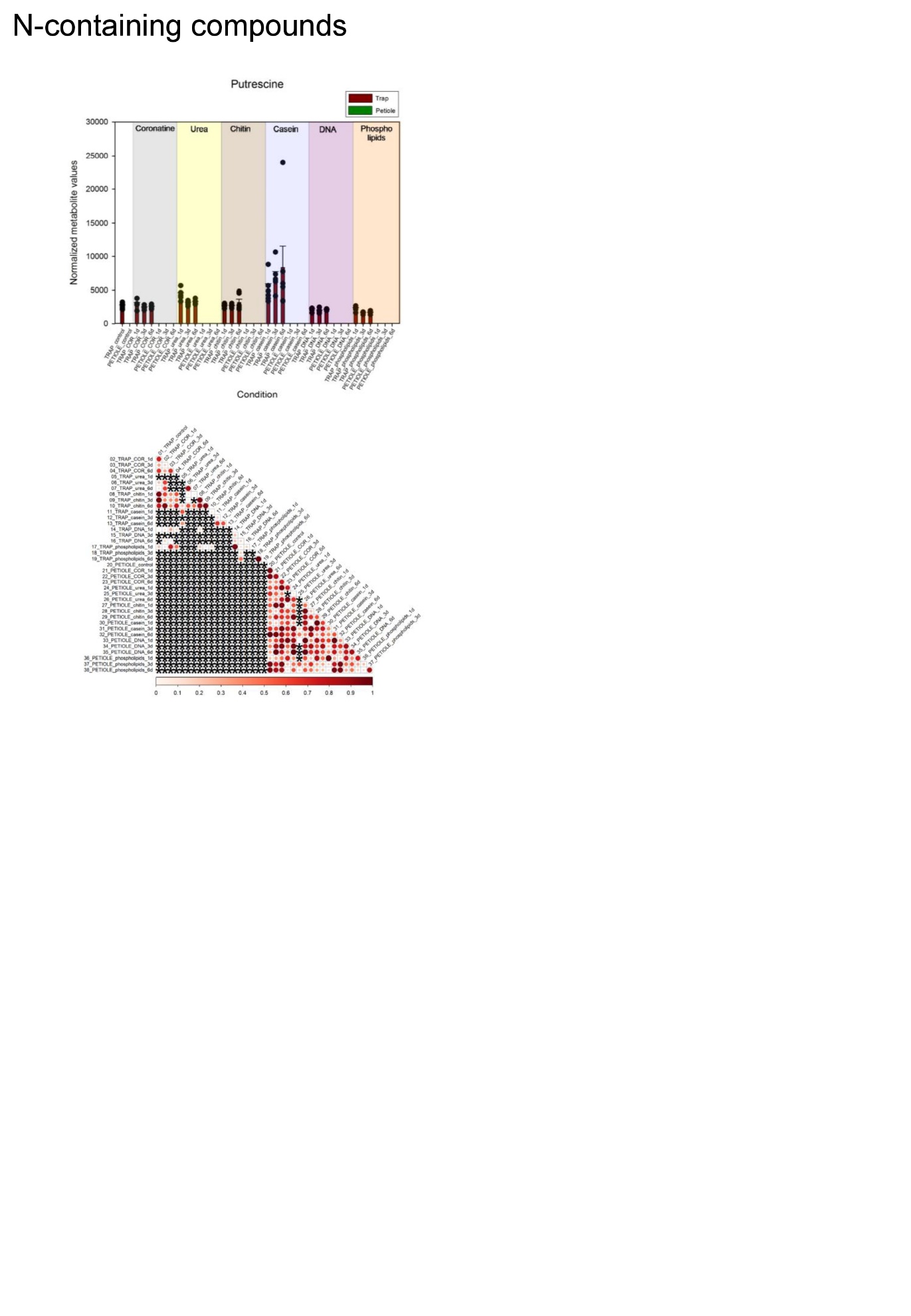

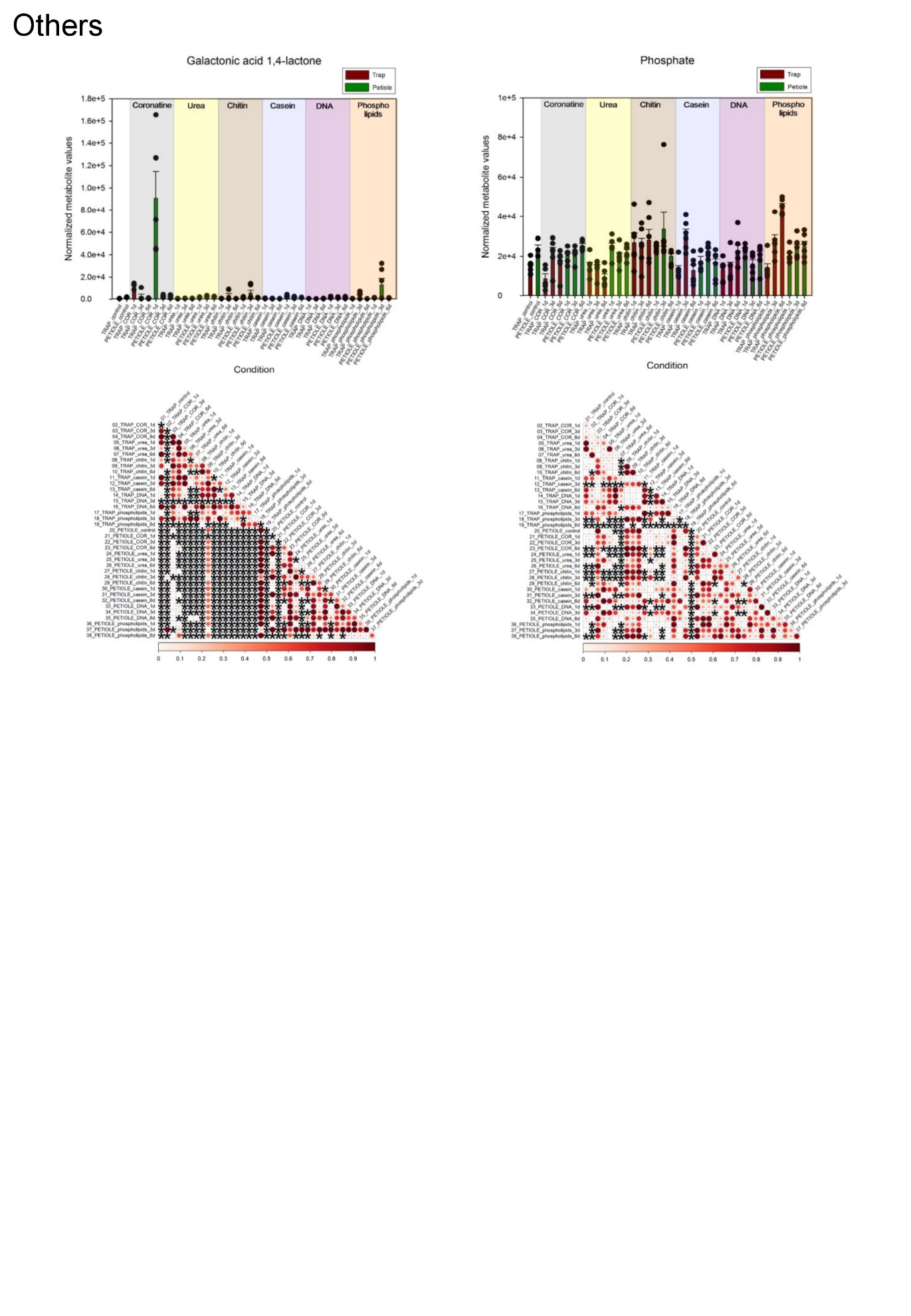


**Fig. S2. Histograms and associated matrices of p-values for the metabolites detected in the substance feeding experiment.** Data are expressed as average normalized metabolite values (a.u.) ± SE. The triangular matrices below each histogram represent the significance of the p-values for all pairwise combinations. An asterisk denotes a significant difference between two conditions (adj pval < 0.05), while red circles denote a non-significant difference (with darker reds for comparisons approaching a p-value of 1).


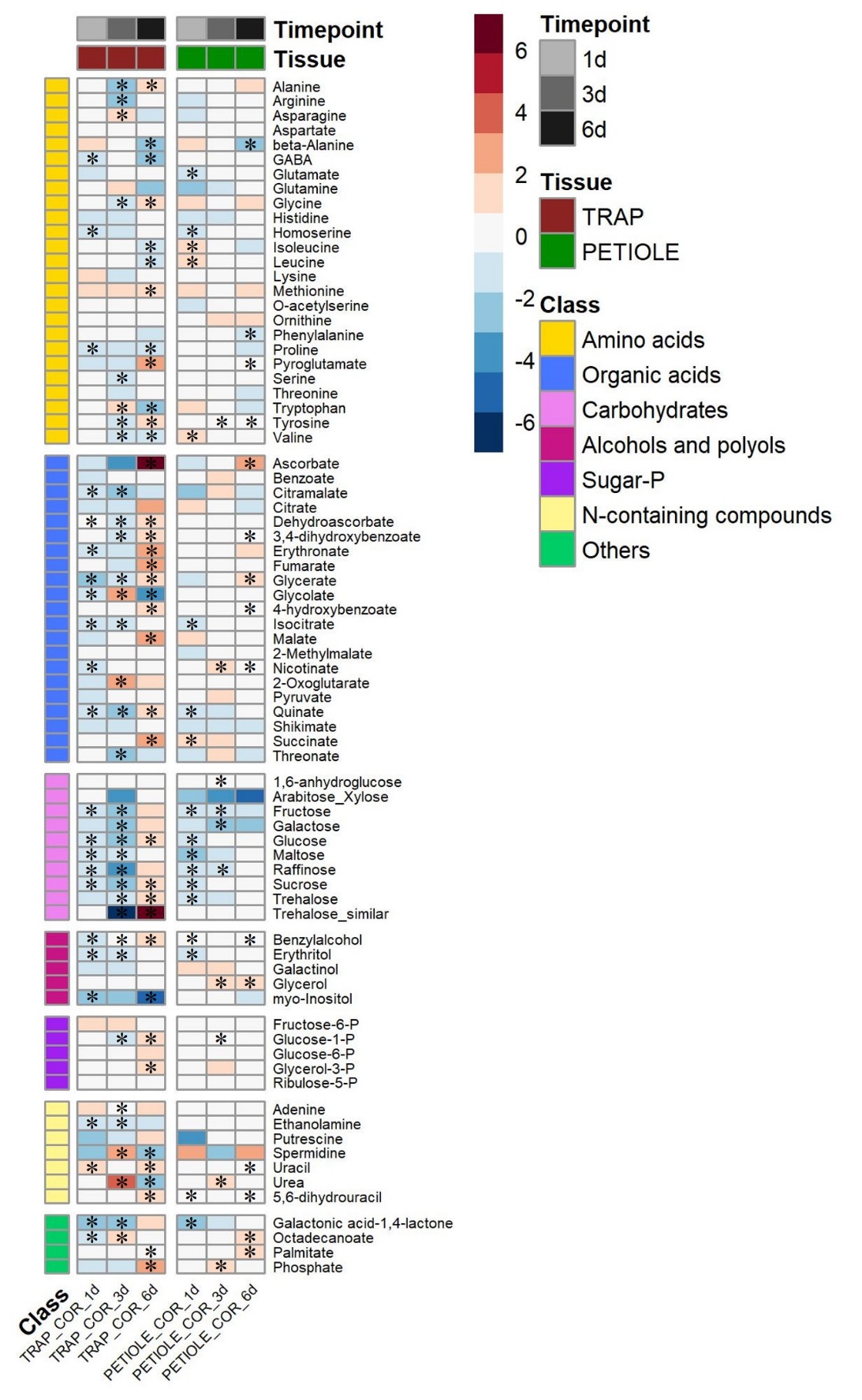


Fig. S3. Changes in metabolite abundance in trap and petiole after inducing trap secretion by spray application of 100 µM coronatine. The heatmap represents the log_2_-fold changes of normalized metabolite abundances in coronatine-treated samples with respect to the mechanostimulated traps and associated petioles at the respective timepoints. Asterisks denote a significant difference between the coronatine-treated sample with respect to its mechanostimulated trap or associated petiole at the respective timepoint (adj pval <0.05, following the initial Kruskal-Wallis test and the Wilcoxon Rank Sum test for multiple comparisons).

Table S1. (separate Excel file)

**Dataset for insect feeding experiment** Table containing metabolite levels measured in mechanostimulated, coronatine and insect-treated traps and petioles at different timepoints after feeding (1, 3 and 6 days). Values represent normalized metabolite levels (by internal standard and weight), in arbitrary units.

Table S2. (separate Excel file)

**Venn diagrams and enrichment analysis**

Excel workbook containing the various steps for construction of Venn diagrams of DE genes and the identification of enriched metabolic pathways for each sector of the Venn diagrams. Enriched pathways were identified using a Chi-square test on a 2x2 contingency table comparing the number of DE genes in a specific metabolic pathway with respect to the DE/non-DE genes detected in all other metabolic pathways.

Table S3. (separate Excel file)

Dataset for substance feeding experiment

Table containing metabolite levels measured in traps and petioles following coronatine treatments and after feeding with urea, chitin, casein, DNA and phospholipids, at different timepoints after feeding (1, 3 and 6 days). Values represent normalized metabolite levels (by internal standard and weight), in arbitrary units.
